# Supplementary material for: Later life outcomes of women by adolescent birth history: analysis of the 2016 Uganda Demographic and Health Survey
Source: BMJ Open. 2021 Feb 10;11(2):e041545. doi: 10.1136/bmjopen-2020-041545 (PMC7878126; doi:10.1136/bmjopen-2020-041545)
Supplement: Supplementary data [file bmjopen-2020-041545supp001.pdf]

Supplementary table 1: Adolescent birth history among women born between 1967-1976 (1995 and 2016 UDHS)

**Adolescent fertility pattern**	**UDHS 1995**	**UDHS 2016**
	N=2,723	N=2,841
	% (95% CI)	% (95% CI)
**No birth <18**	61.0 (58.6-63.5)	63.4 (61.2-65.5)
**Birth <18, no repeat birth <20**	15.0 (13.6-16.5)	14.0 (12.6-15.5)
**Birth <18, repeat birth <20**	24.0 (22.0-26.1)	22.7 (20.9-24.6)
