# Supplementary material for: Later life outcomes of women by adolescent birth history: analysis of the 2016 Uganda Demographic and Health Survey
Source: BMJ Open. 2021 Feb 10;11(2):e041545. doi: 10.1136/bmjopen-2020-041545 (PMC7878126; doi:10.1136/bmjopen-2020-041545)
Supplement: Supplementary data [file bmjopen-2020-041545supp002.pdf]

Supplementary table 2: The variable definitions/categorization for the analysis of the 2016 UDHS

Variable	Categories/definitions
**Residence**	• Rural<br>• Urban
**Region**	• Central (Kampala, Central 1 (South Buganda) & Central 2 (North Buganda))<br>• Eastern (Teso, Karamoja, Bugisu, Bukedi, & Busoga sub-regions)<br>• Northern (Lango, Acholi & West Nile sub-regions)<br>• Western (Bunyoro, Tooro, Ankole & Kigezi sub-regions)
**Religion**	• Anglican (Anglican & Pentecostal/born again/evangelical)<br>• Catholic<br>• Muslim<br>• Other (Seventh day Adventist, orthodox, Baptist, traditional, no religion, other, & Jehovah's Witness)
**Marital status at survey**	• Never in union<br>• Currently in union/marriage (married and living with partner)<br>• Formerly in union/marriage (widowed, divorced, and no longer living together/separated).
**Socioeconomic**	
**Educational attainment**	
Education level	• Incomplete primary (no education & incomplete primary)<br>• Complete primary and higher (Complete primary, incomplete secondary, complete secondary, and higher)
Mean number of years in school (education)	Maintained as a continuous variable
**Literacy**	• cannot read at all (illiterate)<br>• can read (able to read only parts of sentence & able to read whole sentence)<br>• no card with required language<br>• blind/visually impaired
**Receiving cash income at survey**	• Yes (cash only & cash and in-kind)<br>• No (not paid, in-kind only, & missing)
