# Supplementary material for: Later life outcomes of women by adolescent birth history: analysis of the 2016 Uganda Demographic and Health Survey
Source: BMJ Open. 2021 Feb 10;11(2):e041545. doi: 10.1136/bmjopen-2020-041545 (PMC7878126; doi:10.1136/bmjopen-2020-041545)
Supplement: Author's manuscript [file bmjopen-2020-041545.draft_revisions.pdf]

BMJ Open is committed to open peer review. As part of this commitment we make the peer review history of every article we publish publicly available.

When an article is published we post the peer reviewers' comments and the authors' responses online. We also post the versions of the paper that were used during peer review. These are the versions that the peer review comments apply to.

The versions of the paper that follow are the versions that were submitted during the peer review process. They are not the versions of record or the final published versions. They should not be cited or distributed as the published version of this manuscript.

BMJ Open is an open access journal and the full, final, typeset and author-corrected version of record of the manuscript is available on our site with no access controls, subscription charges or pay-per-view fees (http://bmjopen.bmj.com).

If you have any questions on BMJ Open's open peer review process please email
info.bmjopen@bmj.com

BMJ Open

Later Life Outcomes of Women by Fertility Pattern during Adolescence: Analysis of the 2016 Uganda Demographic and Health Survey

Journal:	*BMJ Open*
Manuscript ID	bmjopen-2020-041545
Article Type:	Original research
Date Submitted by the Author:	12-Jun-2020
Complete List of Authors:	Amongin, Dinah; Makerere University School of Public Health, Department of Health Policy Planning and Management; Makerere University College of Health Sciences, Department of obstetrics and Gynaecology<br>Kågesten, Anna; Karolinska Institutet, Department of Global Public Health<br>Tunçalp, Özge; World Health Organizations, 4. Department of Sexual and Reproductive Health and Research, including the UNDP/UNFPA/UNICEF/WHO/World Bank Special Programme of Research, Development and Research Training in Human Reproduction (HRP)<br>Nakimuli, A; Makerere University College of Health Sciences, Department of Obstetrics and Gynecology<br>Nakafeero, Mary; Makerere University School of Public Health, Department of Epidemiology and Biostatistics<br>Atuyambe, Lynn ; Makerere University School of Public Health, Department of Community Health and Behavioral Sciences<br>Hanson, Claudia; Karolinska Institute, Department of Global Public Health; London School of Hygiene and Tropical Medicine Faculty of Infectious and Tropical Diseases, Dept of Disease Control<br>Benova, Lenka; Institute of Tropical Medicine, Department of Public Health; London School of Hygiene & Tropical Medicine, Faculty of Epidemiology and Population Health
Keywords:	OBSTETRICS, REPRODUCTIVE MEDICINE, PUBLIC HEALTH, HEALTH ECONOMICS

SCHOLARONE™
Manuscripts

I, the Submitting Author has the right to grant and does grant on behalf of all authors of the Work (as defined in the below author licence), an exclusive licence and/or a non-exclusive licence for contributions from authors who are: i) UK Crown employees; ii) where BMJ has agreed a CC-BY licence shall apply, and/or iii) in accordance with the terms applicable for US Federal Government officers or employees acting as part of their official duties; on a worldwide, perpetual, irrevocable, royalty-free basis to BMJ Publishing Group Ltd ("BMJ") its licensees and where the relevant Journal is co-owned by BMJ to the co-owners of the Journal, to publish the Work in this journal and any other BMJ products and to exploit all rights, as set out in our licence.

The Submitting Author accepts and understands that any supply made under these terms is made by BMJ to the Submitting Author unless you are acting as an employee on behalf of your employer or a postgraduate student of an affiliated institution which is paying any applicable article publishing charge ("APC") for Open Access articles. Where the Submitting Author wishes to make the Work available on an Open Access basis (and intends to pay the relevant APC), the terms of reuse of such Open Access shall be governed by a Creative Commons licence – details of these licences and which Creative Commons licence will apply to this Work are set out in our licence referred to above.

Other than as permitted in any relevant BMJ Author's Self Archiving Policies, I confirm this Work has not been accepted for publication elsewhere, is not being considered for publication elsewhere and does not duplicate material already published. I confirm all authors consent to publication of this Work and authorise the granting of this licence.

For peer review only

Later Life Outcomes of Women by Fertility Pattern during Adolescence: Analysis of the 2016 Uganda Demographic and Health Survey

Dinah Amongin[1,2*], Anna E. Kågesten[3], Özge Tunçalp[4], Annettee Nakimuli[2], Mary Nakafeero[5], Lynn M. Atuyambe[6], Claudia Hanson [3,7], Lenka Benova[8,9]

1. Department of Health Policy Planning and Management, Makerere university School of Public Health
2. Department of Obstetrics and Gynaecology, School of Medicine, Makerere University College of Health Sciences
3. Department of Global Public Health, Karolinska Institutet, Sweden
4. Department of Sexual and Reproductive Health and Research, including the UNDP/UNFPA/UNICEF/WHO/World Bank Special Programme of Research, Development and Research Training in Human Reproduction (HRP), World Health Organization, Switzerland
5. Department of Epidemiology and Biostatistics, Makerere University School of Public Health
6. Department of Community Health and Behavioral Sciences, Makerere University School of Public Health
7. Department of Disease Control, London School of Hygiene & Tropical Medicine, UK
8. Faculty of Epidemiology and Population Health, London School of Hygiene and Tropical Medicine
9. Department of Public Health, Institute of Tropical Medicine, Antwerp Belgium

***Corresponding author:**

Email**:** amongdinah2003@yahoo.com

Tel: +256704270097

Fax: 256 414 531807

For peer review only

For peer review only - http://bmjopen.bmj.com/site/about/guidelines.xhtml

Abstract

Objectives: We described the long-term socio-economic and reproductive health outcomes of women in Uganda based on adolescent childbearing (initiation <18 and repeat birth <20 years).

Design: Cross-sectional study

Setting: Uganda

Participants: Women aged 40-49 years at the 2016 Uganda Demographic and Health Survey.

Outcome measures: We compared those with first birth <18 years versus not. Among those with a first birth <18, we compared those with and without repeat adolescent births (another birth <20 years). We stratified analysis by marital status at the time of survey. We used two-sample test for proportions, linear regression, and Poisson regression.

Findings

Among the 2,814 women ages 40-49 analyzed, 36.2% reported a first birth <18 years and 85.9% of these had a repeat adolescent birth. Compared to women with no birth <18 years, those with first birth <18 years were less likely to have completed primary education (16.3% versus 32.2%, p<0.001), more likely to be illiterate (55.0% versus 44.0%, p<0.001), to report challenges seeking healthcare (67.6% versus 61.8%, p=0.002), and had higher mean number of births by age 40 years (6.6 versus 5.3, p<0.001). Among the women married at time of survey, those with birth <18 years had older husbands (p<0.001) who also had lower educational attainment (p<0.001). Women without repeat adolescent birth had better socioeconomic and reproductive outcomes compared to those with repeat adolescent birth. Educational attainment, household wealth, total number of births, and under-five mortality among women without repeat adolescent birth were similar, and sometimes better, than among those with no birth <18 years.

Conclusions

For peer review only

Early birth and repeat adolescent births adversely affect life-long socio-economic and health outcomes of women in Uganda. Our results might be birth-cohort specific, but they underscore the need to prevent unwanted adolescent childbearing; with a focus on access to contraceptive services and school continuation.

Strengths and limitations of this study

- Information on later life (40-49 years) outcomes following first birth <18 years in LMIC is limited and the outcomes following repeat adolescent birth (a second or higher birth <20 years following first birth <18 years of age) in sub-Sahara Africa, Uganda inclusive, has not been examined to date.
- Uganda is the first country (and focus of our manuscript), but this analysis and results, using nationally representative data, can be applied to other LMICs.
- The cross sectional nature of the data, Demographic and Health Survey data, does not allow for causal inference and may also be affected by reporting bias from the respondents.
- The results may largely apply to this cohort due to societal changes and advancements in protection of women but, this is a critical starting point to examine long term impact of early birth and repeat adolescent birth.

Funding: This work was supported through the Developing Excellence in Leadership, Training and Science (DELTAS) Africa Initiative grant # DEL-15-011 to THRiVE-2. The DELTAS Africa Initiative is an independent funding scheme of the African Academy of Sciences (AAS)'s Alliance for Accelerating Excellence in Science in Africa (AESA) and supported by the New Partnership for Africa's Development Planning and Coordinating Agency (NEPAD Agency) with funding

For peer review only

For peer review only - http://bmjopen.bmj.com/site/about/guidelines.xhtml

from the Wellcome Trust grant # 107742/Z/15/Z and the UK government. The views expressed in this publication are those of the author(s) and not necessarily those of AAS, NEPAD Agency, Wellcome Trust or the UK government.

Competing interests: None declared.

For peer review only

Introduction

The sub-Saharan Africa region continues to have the highest burden of adolescent births in the world, accounting for 95% of the twelve million births occurring to 15-19-year-old girls each year globally (1-5). Just as in other parts of sub-Sahara Africa, the levels of adolescent childbearing in Uganda remain unacceptably high- 25% of the women age 15-19 years have begun childbearing (are pregnant or have given birth at the point of interview)- , levels similar to 15 years ago (6-9). According to the 2016 Uganda Demographic and Health Survey (UDHS), 28.4% of women aged 20-24 years had a live birth before age 18 years down from 41.7% in 1988/89 survey(10). Among the women 20-24 years who had first birth <18 years, the percentage reporting repeat adolescent birth (first birth <18, and another birth <20 years) had not declined in 30 years (58.9% in 1988/89 versus 55.6% in 2016.

Adolescence is characterized by rapid physical, social, cognitive and emotional development that lay the foundation for an individual's health and wellbeing across the life course. As noted by Patton et al (2018), adolescence is a period when individuals acquire both health, social, cultural, financial and educational assets, whose effects can spill over to the next generation (11). Childbirth

For peer review only - http://bmjopen.bmj.com/site/about/guidelines.xhtml

during adolescence can severely disrupt girls' health and developmental trajectories. Global evidence shows that childbirth in adolescence, especially before age 18, is associated with poor social, economic and health outcomes for both the girl and her offspring (12-16). The health challenges associated with adolescent birth include obstructed labor, postpartum hemorrhage, preterm birth, fistulae, sepsis, preterm birth, and infant death among others (5, 12, 17). In addition, adolescent childbirth can negatively affect socio-economic outcomes leading to school dropout, limited cash income, and forced/early marriage (18-21). Younger adolescent girls are more prone to the negative outcomes due to their immature physiological state, poor economic independence and developing cognition (22, 23). Having repeat pregnancies during adolescence can further exacerbate the risk of such negative outcomes (24, 25).

However, there is limited evidence of the long-term socio-economic and reproductive health outcomes following early initiation of childbearing, including the effects of repeat adolescent birth, in LMICs (26). The available information, mainly from high-income countries, suggests a life-long persistence of socio-economic disadvantage such as low educational attainment, low earnings, and less stable marriages following an adolescent birth (27-30). These studies did not disaggregate outcomes among those who had repeat adolescent birth following the first birth <18 years. They did not explore other outcomes related to reproductive health.

In response to this gap, this paper sought to investigate the socio-economic and reproductive outcomes among women in Uganda toward the end of reproductive life-course (40-49 years) according to their adolescent fertility patterns (no birth <18 years, first birth <18 years, repeat births < 20 years). We hypothesize that initiating childbirth before age 18 leads to lifelong

For peer review only - http://bmjopen.bmj.com/site/about/guidelines.xhtml

For peer review only

disruption in the acquisition of socio-economic resources and wellbeing. Further, we hypothesize that repeat adolescent births (first birth < age 18 and second < age 20) further worsens these outcomes. Given the link between early marriage and childbirth and its potential influence on later life outcomes (28), we assessed outcomes among women with first birth <18 years (comparing those with and without repeat adolescent birth) stratified by marital status at the time of first birth.

Methods

Data sources and population

We used data from the 2016 UDHS individual women's dataset. The study population was women who were aged 40-49 years at the time of the survey. We categorized women age 40-49 years at survey into three adolescent fertility patterns: 1) no birth <18 years; 2) first birth <18 years and no additional births <20 years; and 3) first birth <18 years and one or more additional births <20 years (repeat adolescent birth). We included live births rather than pregnancies and analyzed all women, including those who eventually never gave birth, because, not all pregnancies end in live births; some may end in abortion and others in stillbirths and therefore, the later life outcomes may not be impacted by this. To validate women's recall of adolescent childbearing, we conducted a sensitivity analysis comparing the fertility patterns of women born in 1967-1976 in the 1995 Uganda DHS to the same birth cohort of women in 2016 (Supplementary file 1). We found similar estimates of first birth <18 years and repeat and no repeat adolescent births, indicating similar reporting of fertility patterns by women from the same birth cohort in 1995 versus 2016.

Study variables

For peer review only - http://bmjopen.bmj.com/site/about/guidelines.xhtml

Using the 2016 UDHS, we assessed two main categories of later life outcomes for women with and without adolescent childbirths: socio-economic and reproductive health related outcomes. Supplementary file 2 contains the different variables assessed and their categorization/definitions.

Socio-economic outcomes included women's educational attainment and literacy, household wealth score, being the head of their household, women's occupation and cash income at time of the 2016 UDHS. Educational attainment was assessed as education level and mean number of years in education. Household wealth scores in the DHS are calculated using the household assets and presented as a linear index (31).

Reproductive health outcomes included: i) number of live births by exact age 40 years, ii) number of live births between ages 20 and 40 years, iii) under-five (U5) mortality rate among women's children (any child), iv) unmet need for contraception at the time of survey, v) use of a modern family planning method at the time of survey, vi) number of lifetime sexual partners, vii) condom use at last sex with most recent partner in 12 months preceding the survey, viii) report of a sexually transmitted infection (STI) in 12 months preceding the survey, ix) ever having been tested for HIV, and x) wantedness of the last child (was the child wanted then, wanted later, and wanted no more). Other outcomes linked to reproductive health at time of the 2016 UDHS included women's body mass index category (BMI) and report of challenge seeking healthcare.

Stratification by marital status

For peer review only - http://bmjopen.bmj.com/site/about/guidelines.xhtml

Among women who were married or in a union at the time of the 2016 survey, we analyzed outcomes measured only among this subsample, including age difference with husband; husband's education level (categorized in the same way as for women); husband's occupation; whether the women had been married more than once; and several measures of empowerment: women's response to whether 1) a husband is justified to beat wife, 2) they are part of a marriage with other wives (polygamy), and 3) they are not part of decisions about: a) how savings are spent (for only women who earn money), b) major household purchases, c) visits to family or relatives, d) their own health care, and e) what to do with money husband earns (includes "partner alone" or "other" and excludes those for whom husband lacks earnings).

Background characteristics

We included women's area of residence (urban or rural), geographic region (Central, Eastern, Northern and Western), religion (Anglican, Catholic, Muslim, Other), marital status at survey (currently married/in union, divorced/separated, never in union), and the mean age (years) at survey, at first sex, at first birth, and at first union if ever married/in union.

Statistical analysis

We calculated the percentage of women with different adolescent fertility patterns; with and without first birth <18 years and those with and without repeat adolescent birth (<20 years). Column percentages of the later life outcomes and their 95% confidence intervals for each category of adolescent fertility patterns were presented. The two-sample test of proportions was used to test for differences in the two column proportions for each late life outcome and the respective p-values are provided. For continuous variables, linear regression was used to compare the group means.

For peer review only - http://bmjopen.bmj.com/site/about/guidelines.xhtml

The under-five mortality rates were compared using Poisson regression, with p-values for incidence risk ratios (IRR) presented. The different life course outcomes by fertility pattern were initially described among all the women and separately among the subsample of married women (married/in a union) at survey. We first compared outcomes among women with and without first birth <18 years. We also compared outcomes among those with first birth <18 years who went on to have a repeat adolescent birth by age 20, versus not. For variables with p-value <0.05 between women with and without repeat adolescent births, we analyzed outcomes based on marital status at the time of first birth <18 years. We used survey weights, stratification and clustering to adjust for the complex survey design and non-response. All analyses were conducted using STATA version 12.0, StataCorp LP, Texas.

Missing data

Among the women with first birth <18 years (N=1020), information for marital status at first birth were missing for N=95 (N=10 among those with no repeat adolescent birth and N=85 among those with repeat adolescent birth). We excluded these observations from the analysis by marital status at first birth.

Patient and public involvement

Due to the high adolescent birth in Uganda, the Ministry of Health Uganda was undertaking a landscape analysis of the adolescent health needs but they had a gap in information on the long term outcomes of women by fertility patterns, specifically following repeat adolescent birth. Attention had not been drawn to the women who have another live birth <20 years, following first live birth <18 years. The Reproductive Health Division of the Ministry of Health was involved in setting the objectives and design of the study. The results are to be disseminated to the wider

For peer review only - http://bmjopen.bmj.com/site/about/guidelines.xhtml

stakeholders. For this study, we utilized secondary data and there was no recruitment of participants by the research team.

Ethics

We obtained permission to use the DHS datasets from the DHS program. During the survey process, written informed consent from all the respondents was obtained prior to data collection.

Results

Out of the 2,814 women aged 40-49 years in the 2016 UDHS, 36.2% had a first birth <18 years (Table 1). Among those who had a first birth <18 years, almost nine in ten (85.9%) reported a repeat adolescent birth (31.1% of all women). Among married women, 36.4% reported a first birth <18 years and of these, 87.1% reported a repeat adolescent birth (31.7% of all married women); proportions that were similar to those for the entire sample of women 40-49 years of age.

Table 2 shows the background characteristics of women based on their adolescent fertility patterns. Most of the women in the 2016 sample were from rural areas (79.1%) and of the two main Christian religious faiths (Catholic and Anglican, 86.6%); only 2% had never been married or in a union. Women with and without first birth <18 years had similar distribution of background characteristics in terms of region, residence, religion, marital status, and mean age at survey. However, the mean age at first sex, first birth and first union differed between the groups. Women with first birth <18 had earlier sexual debut (14.4 years vs. 17.3 years, $p<0.001$), with their first birth and union following one versus two years later, respectively (at age 15.5 and 16.3 years). In

For peer review only

For peer review only - http://bmjopen.bmj.com/site/about/guidelines.xhtml

contrast, those with no birth <18 years had their first birth on average four years after first sex and their first union one year before giving birth (21.1, and 20.3 years, respectively). Among women with first birth <18 years, those with repeat adolescent birth entered into marriage or union around the same age (15.9 years) as their first birth (15.5 years) on average, whereas for those without repeat adolescent birth, first marriage or union (18.3 years) happened on average two years after their first birth (16.1 years).

Later life outcomes by fertility patterns

Looking at women all women aged 40-49 years with and without first birth <18 years of age, results from bivariate analysis indicated that women who had their first birth before age 18 had significantly lower mean years of schooling (3.6 vs. 5.0 years, p<0.001), were less likely to only have completed primary school or higher (16.3% versus 32.2%, p<0.001), and more likely to be illiterate (55% vs 44%, p<0.001) compared to those without a birth <18 years (Table 3, Supplementary file 3). Further, they also had lower household wealth score (p=0.043), were more likely to report a challenge seeking health care (p=0.002), and had a higher mean number of live births by exact age 40 years (6.6 versus 5.3 births, p<0.001). However, the mean number of births between age 20-40 years was not substantially different between the two groups (p=0.077). There were no differences between the two groups in children's under-five mortality rate, mean number of lifetime sexual partners, or BMI category.

Among the 1,020 women with first birth <18 years, those with repeat adolescent birth (N=876) were less likely to have completed primary or higher education compared to those without repeat adolescent birth (14.2% vs. 28.9%, p<0.001), Table 3. Further, compared to those without repeat

For peer review only - http://bmjopen.bmj.com/site/about/guidelines.xhtml

adolescent birth, women with a repeat adolescent birth had lower mean years of education (3.4 vs. 4.7 years, p<0.001) and household wealth scores (-0.003 vs. 0.020, p=0.032), were more likely to be illiterate (57.1% vs. 41.9%, p<0.001) and to have had a higher mean number of live births by exact age 40 years (7.0 versus 4.0 live births, p<0.001), including between 20-40 years (5.7 versus 3.0 live births, p<0.001). Further, children born to women with repeat adolescent births had a higher under-five mortality rate than those born to women with no repeat adolescent birth (IRR 1.36, 95% CI 1.06-1.75). Condom use at last sex with most recent partner in last 12 months was nearly twice as high among women with no repeat adolescent birth compared to those with repeat adolescent birth (11.3% vs. 5.6%, p=0.029). Women with and without repeat adolescent birth had similar outcomes in terms of: being head of household, occupation, challenges seeking health care, BMI, unmet need for contraception, mean number of lifetime sexual partners, condom use with most recent partner, self-reported STI in last 12 months, and ever been tested for HIV.

Sub-analysis among women 40-49 married at time of 2016 survey

We further conducted a sub-analysis among women who were married at time of the 2016 UDHS (N=2,022). Compared to married women with no birth <18 years, those with first birth <18 years had higher mean age difference with partner/husband (7.6 versus 5.4 years, p<0.001), percentage of husbands with primary and higher education level (61.2% versus 54.1%, p=0.002), and justification of wife beating (p=0.011) (Table 4). Further, more had control over how their own earnings are spent (p<0.001) although fewer were included in decisions on what to do with the money earned by their husband (p=0.035). There were no differences in outcomes regarding husband's occupation, marriage more than once, polygamous marriage, and non-inclusion in decisions about household purchases, visits to family or relatives, and own health care.

For peer review only - http://bmjopen.bmj.com/site/about/guidelines.xhtml

Among the 737 married women who had a first birth <18 years, those with repeat adolescent birth were less likely to have a husband with a professional occupation compared to those with no repeat adolescent birth (43% vs. 54%, p=0.043). No other differences were observed when comparing married women with and without repeat adolescent birth.

Sub-analysis of women with and without repeat adolescent births, by marital status at first birth (2016 UDHS)

We also conducted a sub-analysis of later life outcomes among women with and without repeat adolescent birth by their marital status at first birth, including variables that were significantly associated with repeat adolescent birth (p<0.05) among all women aged 40-49 years (see Table 3). Of the 791 women with repeat adolescent birth, 76.4% reported being married at first birth compared to 63.4% of those without repeat adolescent birth (p=0.001) (Table 5). As shown in Table 5, there were no differences in later life outcomes based on marital status among women without repeat adolescent births. However, among women with repeat adolescent birth, those who were married at first birth had lower mean years of schooling (3.2 vs. 3.9 years, p=0.017) and were more likely to be illiterate compared to those who were not married at the time (59.7% vs. 49.3%, p=0.012).

Discussion

We investigated the socio-economic and reproductive health outcomes among Ugandan women towards the end of their reproductive life, 40-49 years, using data from the 2016 UDHS. The comparison of outcomes was made between three categories of women; 1) those with and

For peer review only - http://bmjopen.bmj.com/site/about/guidelines.xhtml

297 without first birth <18 years, and 2) among those with first birth <18 years, those with and with no
298 repeat adolescent births (two or more births before age 20). We also conducted sub-analyses to
299 examine differences by marital status both at time of the 2016 survey, and by marital status at the
300 point of the first birth <18 years for those with and with no repeat adolescent birth.

301

302 Our results highlight three key findings. First, women who had their first birth <18 years of age
303 reported more negative socioeconomic and reproductive health outcomes in later life than those
304 without such experiences. These differences appear to be driven by the subgroup of women with
305 repeat adolescent birth who reported lower educational attainment, household wealth scores,
306 empowerment, higher fertility, and higher under-five mortality rates compared to the other groups.
307 Second critical finding is that among women who were married at the time of the 2016 survey,
308 those with repeat adolescent births were also more likely to have husbands who were older and
309 with low educational attainment. Third, we found that women with repeat adolescent birth who
310 were married at time of their first birth <18 years were more likely to be socio-economically
311 disadvantaged in later life compared to never-married peers, particularly related to educational
312 achievement. In contrast, women without repeat adolescent birth had similar, and in some cases
313 better, outcomes than those who reported no birth <18 years, including higher household wealth
314 scores, more control over how their earnings are spent, and lower mean number of live births by
315 40 years of age. Below we discuss these findings in more detail.

316

317 Our first finding was that women with no birth <18 years were more likely to report complete
318 primary and higher education compared to those who initiated childbearing before their 18th
319 birthday. Previous findings show that pregnancy is both a risk-factor for, and consequence of,

For peer review only - http://bmjopen.bmj.com/site/about/guidelines.xhtml

poverty and low education (5, 32, 33). Once an adolescent girl has given birth, she needs social, psychosocial and economic support to go back to school and complete her secondary education (5). This suggests that following the first birth <18 years, adolescent girls do not receive support to continue their education following post childbearing, supporting previous findings of school drop-out due to pregnancy (33) and the need to support girls re-entry or continuation of schooling. Our findings further indicate that the risk of school drop-out was greatest for girls who were married early and went on to have repeat adolescent births, compared to their unmarried counterparts. It is possible that married adolescent women with repeat adolescent birth are taken out of school entirely and are not given the opportunity to resume educational activities that had been prematurely halted (33, 34), underscoring the need to prevent early marriage in a country where marriage before age 18 remains common (40%) (35, 36) and thus a risk factor for repeat adolescent birth and its negative later life outcomes.

Secondly, the fact that women with an adolescent birth (repeat births especially), had lower household wealth scores at the end of their reproductive life course, suggests that low educational attainment and high fertility ultimately lowered their chances of economic empowerment. The premature school discontinuation may have resulted in diminished agency at pregnancy prevention, ultimately leading to many children to cater for (many dependents). Having a partner with a low educational attainment and with non-professional occupation may potentially worsen the negative economic situation. Globally, poorer households have low educational attainment and ultimately high fertility (5). An analysis by the Population Council and Women Deliver, using the 2016 UDHS data among women 20-49 years, compared outcome on employment in last year and receipt of cash earnings among women with and with no first birth <18 years (34). Although the

For peer review only

For peer review only - http://bmjopen.bmj.com/site/about/guidelines.xhtml

authors did not examine outcomes among women with repeat adolescent birth, they noted that women with first birth <18 years were economically disadvantaged in the long term. The quality of employment and cash earnings are linked to the educational attainment, literacy, and status of the spouse which in turn, directly impact on the household wealth score and empowerment (34, 37, 38). Whereas their study did not find differences regarding occupation, women with an adolescent birth had more sole control over how their earnings get spent. This suggested that women with an adolescent birth learnt to be self-reliant. Our study results further reinforce this finding; more women with first birth <18 years have sole control over how their cash income is spent. This finding, on control of cash income, provides an avenue for activities/interventions to economically empower these women. Further, our results call for renewed efforts to alleviate household poverty and maintain adolescent girls in school.

Our study results suggest that high total fertility is supported by an early start but importantly, by repeat adolescent births. Further, the under-five mortality rate follows this pattern; children born to women with repeat adolescent births had significantly higher mortality rate than those without (no birth <18 years and no repeat adolescent birth). A high under five mortality is commonly seen in settings with high fertility, poverty and low educational attainment of the mother (39, 40). Although some decline has been registered, Uganda still has a high age specific fertility among women 15-19 years- 132 births per 1,000 women per year in 2016 (9). This calls for concerted efforts to delay initiation of childbearing, prevent child marriage, maintain girls in school, and promote effective contraceptive programs.

For peer review only - http://bmjopen.bmj.com/site/about/guidelines.xhtml

In other LMIC settings, the limited evidence on later life outcomes points to poor economic outcomes following first birth <18 years (30). Using the DHS data from 43 LMICs, the Population Council and Women Deliver report examined long-term economic outcomes related to employment in the past year and receiving cash earnings - “cash only” - for work among women 20-49 years (41). They found poor economic outcomes in the other countries as well- women with first birth <18 years were significantly less likely to be earning cash although there were no differences in employment. These results are further supported by other studies which found that in the long term, teenage birth resulted in: permanent lower educational attainment, high household poverty, more births, and consequently intergenerational disadvantages- including among their offspring (30, 42-44). In our study, a significantly higher proportion of women with repeat adolescent births ended up with negative reproductive health and empowerment outcomes. Among the subsample of married women, the finding of partners who were older and of lower education among women with first birth <18 years is similar to findings from other studies; women with first birth <18 years had partners of lower socioeconomic standing and tended to be older *(5, 27, 30, 34)*. The socioeconomic status of a woman’s partner/spouse has an impact on the household wealth, empowerment of the woman, and total fertility. Results from our study underscore the need to avail adolescent women with opportunities to prevent first and repeat pregnancy and continue/resume school- as evidenced by interventions in higher income settings (5).

Strength and limitations

This study utilized data from a large nationally representative sample containing a variety of variables. However, the cross-sectional data cannot provide explanations for the outcomes and, the reliance on self-reported accounts carry with them reporting and recall bias that may influence

For peer review only

the results. Available literature indicates that adolescent pregnancy is associated with several risk factors that were present before the pregnancy, such as low educational attainment and low socioeconomic status (28, 45). Some of these risk factors may have been present right from early childhood. Due to this reverse causality, we cannot be certain of what came first; the negative outcomes or the childbirth. Nonetheless, this nationally representative survey data is a starting point to investigate later life outcomes in the absence of a prospective cohort in LMIC setting.

Additionally, circumstances and opportunities available to women approximately 20 years ago may have altered and therefore, it may not fully depict the current situation in Uganda. Still, this study provides an initial insight into where the challenge lies: a repeat adolescent birth. Validation of the adolescent fertility patterns in 2016 was conducted using a similar cohort on the 1995 survey, and the patterns were similar. Regarding the under-five mortality rate, we do acknowledge the changing mortality risk over the years, which have impacted on the overall risk and therefore needed to be adjusted for. However, we hypothesized that the women with an early start have persistent socioeconomic challenges that compromise the survival of any under-five child she had, irrespective of her age at that birth. Future qualitative studies need to explore the motivators for no repeat adolescent birth – the potential enablers for these women to end up similar in outcome to the women who did not have a birth <18 years- and a younger category of women, 20-24 years, with no repeat adolescent birth could be investigated to capture current strategies and enablers.

Conclusion

Findings from this study indicate that initiating childbirth before age 18 is associated with low educational attainment, low household wealth score, and higher fertility and under-five

For peer review only - http://bmjopen.bmj.com/site/about/guidelines.xhtml

mortality outcomes later in life among women in Uganda. This association is especially strong among women who had repeat adolescent birth. The study suggests that women who have first birth <18 years and do not proceed to have a repeat adolescent birth, despite being a minority (5%), appear to recover from the effects of early initiation of birth and in some instances perform better than those with no birth <18 years. Further, women with repeat adolescent birth who were married at first birth, have worse outcomes compared to those not married.

Public health interventions are needed to better prevent repeat adolescent birth and its associated negative life-course outcomes via improved access to continued schooling and higher education, and effective contraceptive services. Further, adolescent women should be protected from early marriage. Our findings call for more in-depth research to explore and define what women without repeat adolescent birth did that enabled them to recuperate from the effects of early initiation of birth. Further, qualitative studies are needed to explore motivators and circumstances for repeat adolescent birth in LMICs. Finally, we suggest that secondary level completion among adolescent mothers be investigated further among younger cohorts, especially now that the policy in Uganda, and many other countries in sub-Sahara Africa, permit girls to continue/resume schooling (32).

References:

1. Temmerman M. Adolescent mothers: too young to be neglected. The Lancet Child & Adolescent Health. 2017;1(3):164-6.

2. Timæus IM, Moultrie TA. Teenage Childbearing and Educational Attainment in South Africa. Studies in Family Planning. 2015;46(2):143-60.

For peer review only - http://bmjopen.bmj.com/site/about/guidelines.xhtml

3. Neal S, Matthews Z, Frost M, Fogstad H, Camacho AV, Laski L. Childbearing in adolescents aged 12-15 years in low resource countries: a neglected issue. New estimates from demographic and household surveys in 42 countries. Acta Obstet Gynecol Scand. 2012;91(9):1114-8.

4. Yakubu I, Salisu WJ. Determinants of adolescent pregnancy in sub-Saharan Africa: a systematic review. Reproductive health. 2018;15(1):15.

5. WHO. Adolescent pregnancy. Fact sheet. World Health Organization, 2020 31 January 2020. Report No.

6. Neal S, Ruktanonchai C, Chandra-Mouli V, Matthews Z, Tatem AJ. Mapping adolescent first births within three east African countries using data from Demographic and Health Surveys: exploring geospatial methods to inform policy. Reprod Health. 2016;13(1):98.

7. Neal SE, Chandra-Mouli V, Chou D. Adolescent first births in East Africa: disaggregating characteristics, trends and determinants. Reproductive health. 2015;12(1):13.

8. Wado YD, Sully EA, Mumah JN. Pregnancy and early motherhood among adolescents in five East African countries: a multi-level analysis of risk and protective factors. BMC pregnancy and childbirth. 2019;19(1):59.

9. UBOS. Uganda Demographic and Health Survey 2016. Kampala, Uganda and Rockville, Maryland, USA: UBOS and ICF, 2018 2018. Report No.

10. Amongin D, Nakimuli A, Hanson C. Time trends in and factors associated with repeat adolescent birth in Uganda: Analysis of six demographic and health surveys. 2020;15(4):e0231557.

11. Patton GC, Olsson CA, Skirbekk V, Saffery R, Wlodek ME, Azzopardi PS, et al. Adolescence and the next generation. Nature. 2018;554(7693):458-66.

12. Loredo-Abdala A, Vargas-Campuzano E, Casas-Munoz A, Gonzalez-Corona J, Gutierrez-Leyva CJ. [Adolescent pregnancy: its causes and repercussions in the dyad]. Rev Med Inst Mex Seguro Soc. 2017;55(2):223-9.

For peer review only - http://bmjopen.bmj.com/site/about/guidelines.xhtml

13. Pérez MM, Barquero ML, Sánchez ML, De L, Los Rios Gestoso GTR, Aso CM, et al. CONSEQUENCES OF TEEN PREGNANCY.

14. Pinto ESJL, Surita FG. Pregnancy in Adolescence - A Challenge Beyond Public Health Policies. Rev Bras Ginecol Obstet. 2017;39(2):41-3.

15. Blanc AK, Winfrey W, Ross J. New findings for maternal mortality age patterns: aggregated results for 38 countries. PLoS One. 2013;8(4):e59864.

16. Nove A, Matthews Z, Neal S, Camacho AV. Maternal mortality in adolescents compared with women of other ages: evidence from 144 countries. The Lancet Global Health. 2014;2(3):e155-e64.

17. United Nations DoEaSA, Population Division. World Fertility 2019: Early and later childbearing among adolescent women. 2020.

18. Atuyambe L, Mirembe F, Johansson A, Kirumira EK, Faxelid E. Experiences of pregnant adolescents--voices from Wakiso district, Uganda. Afr Health Sci. 2005;5(4):304-9.

19. Mollborn S. Teenage Mothers Today: What We Know and How It Matters. Child Development Perspectives. 2017;11(1):63-9.

20. Diaz CJ, Fiel JE. The effect (s) of teen pregnancy: Reconciling theory, methods, and findings. Demography. 2016;53(1):85-116.

21. Ranchhod V, Lam D, Leibbrandt M, Marteleto L. Estimating the effect of adolescent fertility on educational attainment in Cape Town using a propensity score weighted regression. 2011.

22. Leftwich HK, Alves MVO. Adolescent Pregnancy. Pediatric Clinics of North America. 2017;64(2):381-8.

23. Chen X-K, Wen SW, Fleming N, Demissie K, Rhoads GG, Walker M. Teenage pregnancy and adverse birth outcomes: a large population based retrospective cohort study. International journal of epidemiology. 2007;36(2):368-73.

For peer review only

For peer review only - http://bmjopen.bmj.com/site/about/guidelines.xhtml

24. Maravilla JC, Betts KS, Alati R. Increased risk of maternal complications from repeat pregnancy among adolescent women. International Journal of Gynecology & Obstetrics. 2019.

25. Maravilla JC, Betts KS, Alati R. Trends in repeated pregnancy among adolescents in the Philippines from 1993 to 2013. Reproductive health. 2018;15(1):184.

26. Fall CH, Sachdev HS, Osmond C, Restrepo-Mendez MC, Victora C, Martorell R, et al. Association between maternal age at childbirth and child and adult outcomes in the offspring: a prospective study in five low-income and middle-income countries (COHORTS collaboration). The Lancet Global Health. 2015;3(7):e366-e77.

27. Lounds Taylor J. Midlife impacts of adolescent parenthood. Journal of family issues. 2009;30(4):484-510.

28. Hoffman SD, Maynard RA. Kids having kids: Economic costs & social consequences of teen pregnancy: The Urban Insitute; 2008.

29. WomenDeliverandThePopulationCouncil:. *Having a Child Before Becoming an Adult: Exploring the Economic Impact in a Multi-Country Analysis*. New York: 2019. Report No.

30. Arceo-Gómez EO, Campos-Vázquez RM. Teenage pregnancy in Mexico: evolution and consequences. Latin american journal of economics. 2014;51(1):109-46.

31. Filmer D, Pritchett LH. Estimating wealth effects without expenditure data—or tears: an application to educational enrollments in states of India. Demography. 2001;38(1):115-32.

32. Watch HR. LEAVE NO GIRL BEHIND IN AFRICA: Discrimination in Education against Pregnant Girls and Adolescent Mothers. 2018.

33. Wodon Q, Nguyen MC, Tsimpo C. Child Marriage, Education, and Agency in Uganda. Feminist Economics. 2016;22(1):54-79.

34. Women Deliver and The Population Council. Having a child before becoming an adult. Exploring the economic impact in a multi-country Analysis. Report. New York: Women Deliver, 2019 2019. Report No.

35. Male C, Wodon Q. Basic profile of child marriage in Uganda. 2016.

For peer review only

For peer review only - http://bmjopen.bmj.com/site/about/guidelines.xhtml

36. Petroni S, Steinhaus M, Fenn NS, Stoebenau K, Gregowski A. New findings on child marriage in sub-Saharan Africa. Annals of global health. 2017;83(5-6):781-90.

37. Rasmussen B, Maharaj N, Sheehan P, Friedman HS. Evaluating the employment benefits of education and targeted interventions to reduce child marriage. Journal of Adolescent Health. 2019;65(1):S16-S24.

38. Kanjilal-Bhaduri S, Pastore F. Returns to education and female participation nexus: Evidence from India. The Indian Journal of Labour Economics. 2018;61(3):515-36.

39. Maghimbi S. High Fertility, High Infant and Child Mortality And the Tragedy of the Commons in Tanzania. Tanzania Journal for Population studies and Development. 2017;21(1).

40. Kaberuka W, Mugarura A, Tindyebwa J, Bishop DS. Factors determining child mortality in Uganda. International Journal of Social Economics. 2017.

41. Wilkie GL, Leung K, Kumaraswami T, Barlow E, Simas TAM. Effects of Obstetric Complications on Adolescent Postpartum Contraception and Rapid Repeat Pregnancy. Journal of pediatric and adolescent gynecology. 2016;29(6):612-6.

42. Azevedo JP, Lopez-Calva LF, Perova E. Is the baby to blame? An inquiry into the consequences of early childbearing: The World Bank; 2012.

43. Tang S, Davis-Kean PE, Chen M, Sexton HR. Adolescent pregnancy's intergenerational effects: Does an adolescent mother's education have consequences for her children's achievement? Journal of Research on Adolescence. 2016;26(1):180-93.

44. Assini-Meytin LC, Green KM. Long-term consequences of adolescent parenthood among African-American urban youth: A propensity score matching approach. Journal of Adolescent Health. 2015;56(5):529-35.

45. Borkowski JG, Farris JR, Weed K. A dolescent motherhood often represents a constellation of risks presentinthe lives of teenagers from early development to their first pregnancy. These cumulative risks, coupled with an offtimed transition to adulthood, often launch negative life trajectories which spill over to influence children’s lives. Opportunities are

For peer review only

For peer review only - http://bmjopen.bmj.com/site/about/guidelines.xhtml

available at several critical life junctures to transform this developmental scenario, for both teen mothers and their children. An awareness of the risk and protective factors present at each juncture, combined with the delivery of theoreticallydriven interventions, will likely reduce the negative consequences to teen mothers, their children, and their involvement with the larger society. In this chapter, we focus on characteristics of. Risk and Resilience: Adolescent Mothers and Their Children Grow Up. 2016.

Table 1: Adolescent fertility patterns among Uganda women aged 40-49 years at the 2016 UDHS

Adolescent fertility pattern	All women<br>N=2,814<br>% (95% CI)	Currently Married/ in union<br>N=2,022<br>% (95% CI)
**No birth <18**	63.8 (61.5-65.9)	63.5 (61.1-65.9)
**Birth <18, no repeat <20**	5.1 (4.2-6.2)	4.7 (3.7-5.9)
**Birth <18, repeat <20**	31.1 (29.0-33.3)	31.7 (29.4-34.1)

For peer review only

Table 2: Background characteristics of Uganda women 40-49 years by adolescent fertility pattern; 2016 UDHS

	**All women 40-49 years** *col% (95% CI)*	**No birth <18 years** *col% (95% CI)*	**1st Birth <18 years** *col% (95% CI)*	**Birth <18 years, no repeat** *col% (95% CI)*	**Birth <18 years, Repeat** *col% (95% CI)*
**Variables**	**N=2,814**	**N=1,795**	**N=1,020**	**N=144**	**N=876**
**Residence**					
Urban	20.9 (18.7-23.3)	21.0 (18.3-23.9)	20.8 (17.7-24.3)	23.7 (16.4-33.1)	20.3 (17.4-23.7)
Rural	79.1 (76.7-81.3)	79.0 (76.1-81.7)	79.2 (75.7-82.3)	76.3 (66.9-83.6)	79.7 (76.3-82.6)
**Region**					
Central	26.5 (22.3-31.1)	24.4) (20.2-29.3	30.1 (24.8-36.0)	31.4 (21.8-42.9)	29.9 (24.3-36.1)
Eastern	30.3 (26.1-34.9)	29.4 (25.1-34.1)	31.9 (26.8-37.6)	30.2 (21.4-40.6)	32.2 (26.9-38.1)
Northern	17.6 (14.6-21.1)	18.3 (15.1-22.1)	16.5 (13.2-20.2)	18.1 (12.3-26.0)	16.2 (12.9-20.0)
Western	25.6 (21.7-29.8)	27.9 (23.5-32.7)	21.5 (17.6-26.0)	20.3 (13.8-28.9)	21.7 (17.6-26.4)
**Religion**					
Anglican	44.6 (42.0-47.2)	44.8 (41.9-47.9)	44.1 (40.1-48.1)	39.4 (30.6-48.9)	44.8 (40.6-49.2)
Catholic	42.0 (39.2-44.9)	43.2 (39.9-46.5)	40.0 (36.2-43.9)	48.0 (38.9-57.2)	38.7 (34.6-43.0)
Muslim	10.5 (8.8-12.6)	9.1 (7.2-11.4)	13.0 (10.4-16.2)	9.2 (5.3-15.7)	13.7 (10.8-17.1)
Other	2.9 (2.2-3.8)	2.9 (2.1-4.0)	2.9 (1.8-4.5)	3.4 (1.2-9.2)	2.8 (1.7-4.5)
**Marital status at survey**					
Never in union	2.0 (1.5-2.7)	2.4 (1.7-3.4)	1.3 (0.7-2.3)	0.3 (0.0-2.0)	1.4 (0.8-2.6)
Currently in union/marriage	71.8 (69.9-73.7)	71.6 (69.1-73.9)	72.3 (69.0-75.4)	66.4 (57.1-74.5)	73.3 (69.8-76.5)
Formerly in union/marriage	26.2 (24.3-28.1)	26.0 (23.7-28.5)	26.4 (23.4-29.7)	33.4 (25.2-42.6)	25.3 (22.1-28.7)
**Mean age at survey (sd)**	43.9 (2.9)	44.1 (2.8)	43.8 (2.9)	43.5 (2.9)	43.9 (2.9)
**Mean age at first sex (sd)**	16.3 (2.8)	17.3 (2.8)	14.4 (1.5)	15.1 (1.4)	14.3 (1.5)
**Mean age at 1st birth (sd)**	19.0 (3.9)	21.1 (3.3)	15.5 (1.5)	16.1 (1.2)	15.5 (1.5)
**Mean age at 1st union, if ever married (SD)**	18.9 (5.3)	20.3 (4.9)	16.3 (5.0)	18.3 (5.8)	15.9 (4.8)

For peer review only

Table 3: Socioeconomic and health-related life course outcomes by adolescent fertility patterns among Uganda women 40-49 years, 2016 UDHS (N=2815)

	All women 40-49 years			Women with first birth <18 years		
	No birth <18 years **(N=1795)**	Birth <18 years **(N=1020)**		No repeat adolescent birth **(N=144)**	Repeat adolescent birth **(N=876)**	
**Variables**	% (95% CI)	% (95% CI)	***p***	% (95% CI)	% (95% CI)	*p*
**Education level**						
Complete primary and above	32.2 (29.3-35.3)	16.3 (13.7-19.2)	<0.001	28.9 (21.2-37.9)	14.2 (11.6-17.3)	<0.001
**Mean years in school (SD)**	5.0 (4.4)	3.6 (3.2)	<0.001	4.7 (3.4)	3.4 (3.1)	0.001
**Literacy**						
Illiterate (cannot read at all)	44.0 (40.9-47.1)	55.0 (50.8-59.1)	<0.001	41.9 (32.7-51.8)	57.1 (52.8-61.4)	<0.001
**Household wealth score**	0.009 (0.100)	0.001 (0.085)	0.043	0.020 (0.099)	-0.003 (0.082)	0.032
**Women who are head of household**	37.2 (34.6-39.8)	40.3 (36.9-43.8)	0.104	46.1 (36.9-55.6)	39.3 (35.6-43.2)	0.123
**Woman's occupation**						
Professional	34.1 (31.0-37.3)	31.0 (27.5-34.7)	0.093	31.5 (23.1-41.5)	30.9 (27.3-34.7)	0.885
Agricultural-self employed	51.0 (47.8-54.3)	53.1 (49.1-57.1)	0.284	48.0 (38.3-57.8)	54.0 (49.8-58.1)	0.181
**Receiving cash income at survey (yes)**	70.0 (67.0-72.8)	70.5 (67.1-73.7)	0.780	71.3 (61.7-79.2)	70.4 (66.7-73.8)	0.826
**Challenge seeking health care (yes)**	61.8 (58.6-64.9)	67.6 (63.9-71.1)	0.002	62.0 (52.2-70.9)	68.5 (64.5-72.3)	0.123
**BMI of woman at survey**	N=585	N=343		N=52	N=291	
Underweight	9.5 (7.3-12.3)	6.7 (4.3-10.2)	0.139	13.1 (6.2-25.4)	5.5 (3.3-9.1)	0.195
Over weight/obese	33.2 (28.8-38.0)	35.0 (29.3-41.1)	0.576	38.9 (24.7-55.2)	34.3 (28.3-40.9)	0.522
**Mean No. of live births by exact age 40 years (SD)**	5.3 (2.1)	6.6 (1.7)	<0.001	4.0 (1.6)	7.0 (1.3)	<0.001
**Mean No. of live births between age 20-<40 years (SD)**	5.1 (2.1)	5.3 (2.1)	0.077	3.0 (1.6)	5.7 (1.9)	<0.001
**U5 mortality rate**	124.6	140.1	P=0.341	104.8	142.9	P=0.016
**Unmet need for contraception**	19.8 [17.8,21.9]	20.2 (17.4-23.4)	0.799	20.1 (13.0-29.6)	20.2 [17.1,23.8]	0.978
**Current use of modern FP**	26.5 (24.3-29.0)	28.9 (25.8-32.2)	0.170	21.4 (14.9-29.7)	30.1 (26.7-33.7)	0.033
**Mean number of lifetime sexual partners (SD)**	2.7 (6.7)	3.5 (10.2)	0.062	2.9 (2.4)	3.6 (10.9)	0.125
**Condom use at last sex with most recent partner**	N=1378	N=759		N=100	N=659	
Yes, used	6.4 (5.0-8.1)	6.4 (4.4-9.0)	1.000	11.3 (5.7-21.2)	5.6 (3.7-8.3)	0.029
**Self-reported STI in last 12 months**	8.4 (6.9-10.3)	7.1 (5.4-9.1)	0.220	10.0 (5.4-17.5)	6.6 (4.8-8.9)	0.216
**Ever been tested for HIV**	91.4 (89.7-92.8)	92.5 (90.5-94.0)	0.307	90.1 (83.5-94.3)	92.9 (90.8-94.5)	0.321

* Under5mortality: Divided by 1000 live births. P-value obtained for the IRR that compared the mortality rate between 2 groups. P=0.016, IRR was 1.36 (95% CI 1.06-0.75).

For peer review only

For peer review only - http://bmjopen.bmj.com/site/about/guidelines.xhtml

Table 4: Later life outcomes related to marital characteristics among a subsample of married women 40-49 (2016 UDHS, N=2022)

	All women 40-49 years			Women with first birth <18 years		
	**No birth <18 years (N=1285)**	**Birth <18 years (N=737)**		**No repeat adol. birth (N=096)**	**Repeat adol. birth (N=642)**	
**Variables**	% (95% CI)	% (95% CI)	*p*	% (95% CI)	% (95% CI)	*p*
**Mean age difference with partner/husband- years (SD)**	5.4 (6.4)	7.6 (7.2)	<0.001	6.8 (7.1)	7.7 (7.2)	0.358
**Husband's education level**	N=1285	N=737		N=96	N=642	
Primary	54.1 (50.5-57.7)	61.2 (56.9-65.3)	0.002	60.0 (48.6-70.5)	61.3 (56.8-65.6)	0.808
Secondary/higher	34.1 (30.7-37.7)	26.6 (22.9-30.6)	<0.001	31.7 (22.2-43.0)	25.8 (22.0-30.0)	0.222
**Husband's occupation**						
Professional	46.2 (42.9-49.6)	44.4 (40.2-48.7)	0.434	54.0 (42.6-65.0)	43.0 (38.4-47.6)	0.043
Agricultural-self employed	44.1 (40.6-47.7)	44.0 (39.6-48.4)	0.965	36.1 (25.7-48.0)	45.1 (40.4-49.9)	0.098
**Women married more than once**	24.0 (21.4-27.0)	26.1 (22.3-30.2)	0.292	26.2 (17.3-37.6)	26.0 (22.2-30.3)	0.967
**Reported that husband is justified to beat a wife for any of the reasons listed (yes)**	45.0 (41.5-48.5)	50.9 (46.7-55.0)	0.011	45.5 (33.5-58.0)	51.7 (47.0-56.2)	0.257
**Women who solely decide on how her earnings are spent**	N=897<br>52.1 (48.3-55.9)	N=499<br>64.2 (58.8-69.3)	<0.001	N=68<br>64.8 (50.2-77.0)	N=499<br>64.1 (58.6-69.3)	0.910
**Women not included on decisions about:**						
Major household purchases	25.1 (22.3-28.1)	28.5 (24.3-33.1)	0.095	31.2 (20.8-43.9)	28.1 (23.9-32.7)	0.530
Visits to family or relatives	20.7 (18.0-23.6)	23.0 (19.8-26.5)	0.226	22.0 (13.8-33.0)	23.2 (19.7-27.0)	0.795
Their own health care	18.8 (16.4-21.4)	20.9 (17.5-24.8)	0.252	15.5 (8.7-26.0)	21.8 (17.9-26.2)	0.157
**What to do with money husband earns**	N=1269<br>48.4 (45.2-51.6)	N=730<br>53.3 (48.9-57.6)	0.035	N=96<br>53.3 (41.0-65.2)	N=634<br>53.3 (48.7-57.8)	1.000
**In marriage with other wives (polygamy)**	33.1 (29.8-36.6)	36.2 (32.3-40.2)	0.157	42.9 (31.1-55.6)	35.2 (31.1-39.4)	0.143

For peer review only

Table 5: Later life comes among women 40-49 years with and without repeat adolescent birth, by marital status at first birth (N=925)

	**No repeat adolescent birth**			**Repeat adolescent birth**		
	**Not married at 1st birth (N=49)**	**Married at 1st birth (N=85)**		**Not married at 1st birth (N=186)**	**Married at 1st birth (N=604)**	
**Variables**	% (95% CI)	% (95% CI)	***p***	% (95% CI)	% (95% CI)	***p***
**Education level**						
Complete primary and above	29.6 (17.5-45.4)	24.7 (15.7-36.5)	0.536	17.4 (12.1-24.4)	12.4 (9.7-15.8)	0.082
**Mean years in school (SD)**	4.7 (3.0)	4.2 (3.2)	0.458	3.9 (3.0)	3.2 (3.1)	0.017
**Literacy**						
Cannot read at all (illiterate)	40.3 (25.2-57.5)	44.4 (32.2-57.4)	0.644	49.3 (40.1-58.6)	59.7 (54.7-64.5)	0.012
**Household wealth scores** (SD)	0.005 (0.082)	0.024 (0.097)	0.320	0.004(0.085)	-0.008 (0.078)	0.097
**Mean No. of live births by exact age 40 years** (39.99) (SD)	4.0 (1.7)	4.2 (1.6)	0.602	7.0 (1.2)	7.0 (1.3)	0.963
**Mean No. of live births between age 20-<40 years (SD)**	3.0 (1.7)	3.2 (1.6)	0.602	5.8 (1.8)	5.7 (1.9)	0.652
**U5 mortality rate**/1000 live births	109.3	107.1	0.884	140.4	144.6	0.803

NB: * P-value of the IRR obtained from comparing the mortality rates. P=0.884 (IRR 0.98, 95%CI=0.75-1.28) and p=0.803 (IRR 1.03, 95% CI=0.82-1.30)

Abbreviations: BMI, Body Mass Index; DHS, Demographic and Health Surveys; FP, Family Planning; IRR, Incidence risk Ratio; LMIC, Low-and-Middle-Income Country; SD, Standard Deviation; STI, Sexually Transmitted Infection; UDHS, Uganda Demographic and Health Survey; U5, Under-five.

Author contributions:

DA conceived and designed the study, obtained permission to use data set from DHS program, conducted analysis, presented and interpreted results, drafted and finalized the article. AK conceived the study, conducted the literature review, reviewed and interpreted the results, and reviewed the article. ÖT conceived the study, drafted the analysis plan, reviewed and interpreted

For peer review only

For peer review only - http://bmjopen.bmj.com/site/about/guidelines.xhtml

the results and reviewed the article. AN was involved reviewing the study design, the results and drafting the article. MN was involved in data analysis, presentation and interpretation of the results. LA was involved in reviewing and interpreting the results and reviewing the manuscript. CH was involved in designing the study, the analysis, presenting and interpreting the results, drafting the article and reviewing the manuscript. LB was involved in conceiving the study, designing the analysis plan, data analysis, interpreting the results and substantially reviewing the article. All the authors reviewed and approved the manuscript. All the authors take responsibility for their contributions. None of the authors has competing interests.

Data sharing: Data used for this analysis can be accessed, with permission, from the Demogrpahic and Health Survey program website https://www.dhsprogram.com/data/available-datasets.cfm

For peer review only

Supplementary table 1: Adolescent fertility patterns- among women born between 1967-1976 (1995 and 2016 UDHS)

**Adolescent fertility pattern**	**UDHS 1995**	**UDHS 2016**
	N=2,723	N=2,841
	% (95% CI)	% (95% CI)
**No birth <18**	61.0 (58.6-63.5)	63.4 (61.2-65.5)
**Birth <18, no repeat birth <20**	15.0 (13.6-16.5)	14.0 (12.6-15.5)
**Birth <18, repeat birth <20**	24.0 (22.0-26.1)	22.7 (20.9-24.6)

For peer review only

Supplementary table 2: The variable definitions/categorization for the analysis of the 2016 UDHS

Variable	Categories/definitions
**Residence**	• Rural<br>• Urban
**Region**	• Central (Kampala, Central 1 (South Buganda) & Central 2 (North Buganda))<br>• Eastern (Teso, Karamoja, Bugisu, Bukedi, & Busoga sub-regions)<br>• Northern (Lango, Acholi & West Nile sub-regions)<br>• Western (Bunyoro, Tooro, Ankole & Kigezi sub-regions)
**Religion**	• Anglican (Anglican & Pentecostal/born again/evangelical)<br>• Catholic<br>• Muslim<br>• Other (Seventh day Adventist, orthodox, Baptist, traditional, no religion, other, & Jehovah's Witness)
**Marital status at survey**	• Never in union<br>• Currently in union/marriage (married and living with partner)<br>• Formerly in union/marriage (widowed, divorced, and no longer living together/separated).
**Socioeconomic**	
**Educational attainment**	
Education level	• Incomplete primary (no education & incomplete primary)<br>• Complete primary and higher (Complete primary, incomplete secondary, complete secondary, and higher)
Mean number of years in school (education)	Maintained as a continuous variable
**Literacy**	• cannot read at all (illiterate)<br>• can read (able to read only parts of sentence & able to read whole sentence)<br>• no card with required language<br>• blind/visually impaired
**Occupation**	• Professional (professional/technical/managerial, clerical, sales, services, & skilled manual)<br>• Agriculture-self-employed<br>• Not working (not working, household and domestic, unskilled manual, & missing)<br>• Other occupations (household and domestic, unskilled manual, & missing)
**Receiving cash income at survey**	• Yes (cash only & cash and in-kind)<br>• No (not paid, in-kind only, & missing)
**BMI of women at survey**	• Underweight (<18.49 kg/m2),<br>• Normal (18.50-24.99 kg/m2)<br>• Overweight/obese (>25.00 kg/m2)
**Extra variables in the supplementary table 2**	
**Education level-detail**	• No education<br>• Incomplete primary<br>• Complete primary<br>• Incomplete secondary<br>• Complete secondary & higher
**Literacy**	• Cannot read at all<br>• Able to read only parts of sentence (Reads part of sentence)<br>• Able to read whole sentence<br>• Other (no card with required language, blind/visually impaired)
**Household wealth quintile**	• Poorest<br>• Poorer<br>• Middle<br>• Richer<br>• Richest

For peer review only

For peer review only

Supplementary table 3: Detailed table of different life-course outcomes by fertility patterns among Uganda women 40-49 years, UDHS 2016 (N=2814)

Variables	No birth <18 years N= 1795 %(95% CI)	Birth <18 years N= 1020 %(95% CI)	*p*	Birth <18 years, no repeat N= 144 %(95% CI)	Birth <18 years, Repeat N= 876 %(95% CI)	*p*
Educational achievement						
**Education level-detail**						
No education	21.1 (18.8-23.7)	27.4 (24.2-30.9)	<0.001	15.7 (10.1-23.5)	29.3 (25.8-33.2)	<0.001
Incomplete primary	46.7 (43.8-49.6)	56.3 (52.7-59.8)	<0.001	55.5 (46.3-64.3)	56.5 (52.5-60.3)	0.823
Complete primary	11.6 (9.9-13.5)	8.9 (7.1-11.0)	<0.001	16.6 (10.9-24.4)	7.6 (5.8-9.9)	<0.001
Incomplete secondary	12.5 (10.7-14.5)	5.9 (4.4-7.9)	<0.001	9.1 (5.0-16.1)	5.4 (3.9-7.5)	0.081
Complete Secondary/higher	8.2 (6.5-10.2)	1.5 (0.8-2.8)	<0.001	3.2 (1.0-9.3)	1.2 (0.6-2.3)	0.066
**Literacy**						
Cannot read at all	44.0 (40.9-47.1)	55.0 (50.8-59.1)	<0.001	41.9 (32.7-51.8)	57.1 (52.8-61.4)	<0.001
Reads part of sentence	10.6 (8.9-12.5)	10.3 (8.2-12.7)	0.803	10.2 (5.7-17.6)	10.3 (8.1-12.9)	0.971
Able to read	44.2 (41.3-47.1)	33.3 (29.6-37.1)	<0.001	45.8 (36.0-56.0)	31.2 (27.5-35.1)	<0.001
Other	1.3 (0.8-2.0)	1.5 (0.8-2.7)	0.661	2.0 (0.5-8.2)	1.4 (0.7-2.7)	0.581
**Household wealth quintile at survey**						
poorest	18.7 (16.1-21.7)	15.9 (13.5-18.7)	0.061	16.9 (0.7-2.7)	15.7 (13.1-18.7)	0.715
poorer	17.7 (15.8-19.8)	20.3 (17.7-23.2)	0.089	15.5 (10.1-23.1)	21.1 (18.3-24.2)	0.122
middle	21.3 (19.0-23.7)	24.0 (21.0-27.2)	0.098	19.3 (12.7-28.2)	24.7 (21.5-28.3)	0.159
richer	21.6 (19.3-24.1)	24.2 (21.1-27.5)	0.113	26.5 (18.8-35.8)	23.8 (20.4-27.5)	0.483
richest	20.7 (18.0-23.7)	15.7 (12.9-18.9)	0.001	21.9 (14.9-31.0)	14.7 (11.9-18.0)	0.028
**Woman's occupation**						
Not working	10.4 (8.6-12.5)	10.7 (8.6-13.2)	0.803	13.3 (7.6-22.4)	10.2 (8.1-12.9)	0.264
Professional	34.1 (31.0-37.3)	31.0 (27.5-34.7)	0.093	31.5 (23.1-41.5)	30.9 (27.3-34.7)	0.885
Agricultural-self employed	51.0 (47.8-54.3)	53.1 (49.1-57.1)	0.284	48.0 (38.3-57.8)	54.0 (49.8-58.1)	0.181
Other occupations	4.5 (3.2-6.2)	5.2 (3.6-7.5)	0.402	7.1 (3.2-15.1)	4.9 (3.3-7.3)	0.271
**Empowerment**						
**Access to health care: % of women who said;**						
**Getting** permission to go is a big problem when seeking health care for themselves	3.8 (3.0-5.0)	4.1 (3.0-5.7)	0.693	3.5 (1.3-9.1)	4.2 (3.0-5.9)	0.695
**Getting** money to go is a big problem when seeking health care for themselves	51.1 (47.9-54.3)	56.9 (53.2-60.5)	0.003	51.9 (42.4-61.2)	57.8 (53.7-61.7)	0.185
**Distance** to facility is a big problem when seeking health care for themselves	41.0 (37.9-44.1)	44.4 (40.4-48.4)	0.079	44.6 (34.9-54.8)	44.3 (40.1-48.7)	0.946
**Not** wanting to go alone is a big problem when seeking health care for themselves	21.1 (18.8-23.6)	21.7 (18.9-24.8)	0.709	22.4 (15.4-31.4)	21.5 (18.5-24.9)	0.808
**Husband is justified in beating a wife if;**						
She goes out without telling husband	27.2 (24.8-29.7)	30.4 (27.2-33.9)	0.070	27.5 (19.7-36.9)	30.9 (27.2-34.9)	0.411
wife neglects children	33.3 (30.6-36.2)	38.4 (35.0-41.9)	0.006	33.7 (25.2-43.5)	39.2 (35.4-43.1)	0.209
wife argues with husband	23.5 (21.2-26.0)	26.9 (23.6-30.4)	0.045	23.5 (16.5-32.3)	27.4 (24.0-31.2)	0.328
wife refuses husband sex	19.9 (17.8-22.2)	19.7 (17.0-22.7)	0.898	20.1 (13.9-28.0)	19.6 (16.8-22.8)	0.889
she burns food	12.1 (10.4-14.0)	12.2 (10.1-14.8)	0.938	13.5 (8.5-20.7)	12.0 (9.8-14.7)	0.610
RH outcomes						
**% wanted last child**	N=623	N=276	N=899	N=14	N=261	
Wanted then	46.4 (41.9-51.0)	34.0 (28.1-40.6)	<0.001	73.6 (47.3-89.7)	31.9 (25.8-38.6)	0.001
Wanted later	14.7 (11.8-18.1)	9.6 (6.3-14.3)	0.037	6.1 (0.8-33.8)	9.8 (6.4-14.7)	0.647
Wanted no more	38.8 (34.7-43.2)	56.4 (50.0-62.6)	<0.001	20.3 (7.0-46.1)	58.4 (51.7-64.7)	0.005

*****NB: *The BMI information excluded women who were pregnant at the time of the survey.*

For peer review only

STROBE Statement—Checklist of items that should be included in reports of ***cross-sectional studies***

	Item No	Recommendation	Page No
**Title and abstract**	1	(*a*) Indicate the study's design with a commonly used term in the title or the abstract	1
		(*b*) Provide in the abstract an informative and balanced summary of what was done and what was found	3
**Introduction**			
Background/rationale	2	Explain the scientific background and rationale for the investigation being reported	6
Objectives	3	State specific objectives, including any prespecified hypotheses	7
**Methods**			
Study design	4	Present key elements of study design early in the paper	8
Setting	5	Describe the setting, locations, and relevant dates, including periods of recruitment, exposure, follow-up, and data collection	8
Participants	6	(*a*) Give the eligibility criteria, and the sources and methods of selection of participants	8
Variables	7	Clearly define all outcomes, exposures, predictors, potential confounders, and effect modifiers. Give diagnostic criteria, if applicable	9
Data sources/ measurement	8*	For each variable of interest, give sources of data and details of methods of assessment (measurement). Describe comparability of assessment methods if there is more than one group	*9*
Bias	9	Describe any efforts to address potential sources of bias	10
Study size	10	Explain how the study size was arrived at	8
Quantitative variables	11	Explain how quantitative variables were handled in the analyses. If applicable, describe which groupings were chosen and why	9
Statistical methods	12	(*a*) Describe all statistical methods, including those used to control for confounding	10
		(*b*) Describe any methods used to examine subgroups and interactions	9
		(*c*) Explain how missing data were addressed	11
		(*d*) If applicable, describe analytical methods taking account of sampling strategy	11
		(*e*) Describe any sensitivity analyses	8
**Results**			
Participants	13*	(a) Report numbers of individuals at each stage of study—eg numbers potentially eligible, examined for eligibility, confirmed eligible, included in the study, completing follow-up, and analysed	12
		(b) Give reasons for non-participation at each stage	NA
		(c) Consider use of a flow diagram	NA
Descriptive data	14*	(a) Give characteristics of study participants (eg demographic, clinical, social) and information on exposures and potential confounders	12
		(b) Indicate number of participants with missing data for each variable of interest	11
Outcome data	15*	Report numbers of outcome events or summary measures	13
Main results	16	(*a*) Give unadjusted estimates and, if applicable, confounder-adjusted estimates and their precision (eg, 95% confidence interval). Make clear which confounders were adjusted for and why they were included	13

For peer review only

		(*b*) Report category boundaries when continuous variables were categorized	13
		(*c*) If relevant, consider translating estimates of relative risk into absolute risk for a meaningful time period	NA
Other analyses	17	Report other analyses done—eg analyses of subgroups and interactions, and sensitivity analyses	13/14
**Discussion**			
Key results	18	Summarise key results with reference to study objectives	16
Limitations	19	Discuss limitations of the study, taking into account sources of potential bias or imprecision. Discuss both direction and magnitude of any potential bias	19
Interpretation	20	Give a cautious overall interpretation of results considering objectives, limitations, multiplicity of analyses, results from similar studies, and other relevant evidence	20
Generalisability	21	Discuss the generalisability (external validity) of the study results	21
**Other information**			
Funding	22	Give the source of funding and the role of the funders for the present study and, if applicable, for the original study on which the present article is based	2

*Give information separately for exposed and unexposed groups.

Note: An Explanation and Elaboration article discusses each checklist item and gives methodological background and published examples of transparent reporting. The STROBE checklist is best used in conjunction with this article (freely available on the Web sites of PLoS Medicine at http://www.plosmedicine.org/, Annals of Internal Medicine at http://www.annals.org/, and Epidemiology at http://www.epidem.com/). Information on the STROBE Initiative is available at www.strobe-statement.org.

For peer review only

BMJ Open

Later Life Outcomes of Women by Adolescent Birth History: Analysis of the 2016 Uganda Demographic and Health Survey

Journal:	*BMJ Open*
Manuscript ID	bmjopen-2020-041545.R1
Article Type:	Original research
Date Submitted by the Author:	20-Nov-2020
Complete List of Authors:	Amongin, Dinah; Makerere University College of Health Sciences, Department of Health Policy Planning and Management<br>Kågesten, Anna; Karolinska Institutet, Department of Global Public Health<br>Tunçalp, Özge; World Health Organizations, 4. Department of Sexual and Reproductive Health and Research, including the UNDP/UNFPA/UNICEF/WHO/World Bank Special Programme of Research, Development and Research Training in Human Reproduction (HRP)<br>Nakimuli, A; Makerere University College of Health Sciences, Department of Obstetrics and Gynecology<br>Nakafeero, Mary; Makerere University School of Public Health, Department of Epidemiology and Biostatistics<br>Atuyambe, Lynn ; Makerere University School of Public Health, Department of Community Health and Behavioral Sciences<br>Hanson, Claudia; Karolinska Institute, Department of Global Public Health; London School of Hygiene and Tropical Medicine Faculty of Infectious and Tropical Diseases, Dept of Disease Control<br>Benova, Lenka; Institute of Tropical Medicine, Department of Public Health; London School of Hygiene & Tropical Medicine, Faculty of Epidemiology and Population Health
**Primary Subject Heading**:	Obstetrics and gynaecology
Secondary Subject Heading:	Epidemiology, Global health, Health policy, Obstetrics and gynaecology, Sexual health
Keywords:	OBSTETRICS, REPRODUCTIVE MEDICINE, PUBLIC HEALTH, SEXUAL MEDICINE

SCHOLARONE™
Manuscripts

I, the Submitting Author has the right to grant and does grant on behalf of all authors of the Work (as defined in the below author licence), an exclusive licence and/or a non-exclusive licence for contributions from authors who are: i) UK Crown employees; ii) where BMJ has agreed a CC-BY licence shall apply, and/or iii) in accordance with the terms applicable for US Federal Government officers or employees acting as part of their official duties; on a worldwide, perpetual, irrevocable, royalty-free basis to BMJ Publishing Group Ltd ("BMJ") its licensees and where the relevant Journal is co-owned by BMJ to the co-owners of the Journal, to publish the Work in this journal and any other BMJ products and to exploit all rights, as set out in our licence.

The Submitting Author accepts and understands that any supply made under these terms is made by BMJ to the Submitting Author unless you are acting as an employee on behalf of your employer or a postgraduate student of an affiliated institution which is paying any applicable article publishing charge ("APC") for Open Access articles. Where the Submitting Author wishes to make the Work available on an Open Access basis (and intends to pay the relevant APC), the terms of reuse of such Open Access shall be governed by a Creative Commons licence – details of these licences and which Creative Commons licence will apply to this Work are set out in our licence referred to above.

Other than as permitted in any relevant BMJ Author's Self Archiving Policies, I confirm this Work has not been accepted for publication elsewhere, is not being considered for publication elsewhere and does not duplicate material already published. I confirm all authors consent to publication of this Work and authorise the granting of this licence.

For peer review only

Later Life Outcomes of Women by Adolescent Birth History: Analysis of the 2016 Uganda Demographic and Health Survey

Dinah Amongin[1,2*], Anna E. Kågesten[3], Özge Tunçalp[4], Annettee Nakimuli[2], Mary Nakafeero[5], Lynn M. Atuyambe[6], Claudia Hanson [3,7], Lenka Benova[8,9]

1. Department of Health Policy Planning and Management, Makerere University School of Public Health
2. Department of Obstetrics and Gynaecology, School of Medicine, Makerere University College of Health Sciences
3. Department of Global Public Health, Karolinska Institutet, Sweden
4. Department of Sexual and Reproductive Health and Research, including the UNDP/UNFPA/UNICEF/WHO/World Bank Special Programme of Research, Development and Research Training in Human Reproduction (HRP), World Health Organization, Switzerland
5. Department of Epidemiology and Biostatistics, Makerere University School of Public Health
6. Department of Community Health and Behavioral Sciences, Makerere University School of Public Health
7. Department of Disease Control, London School of Hygiene & Tropical Medicine, UK
8. Faculty of Epidemiology and Population Health, London School of Hygiene and Tropical Medicine
9. Department of Public Health, Institute of Tropical Medicine, Antwerp Belgium

***Corresponding author:**

Email**:** amongdinah2003@yahoo.com

Tel: +256704270097

Fax: +256 414 531807

For peer review only - http://bmjopen.bmj.com/site/about/guidelines.xhtml

Abstract

Objectives: To describe the long-term socio-economic and reproductive health outcomes of women in Uganda by adolescent birth history.

Design: Cross-sectional study

Setting: Uganda

Participants: Women aged 40-49 years at the 2016 Uganda Demographic and Health Survey.

Outcome measures: We compared socioeconomic and reproductive outcomes among those with first birth <18 years versus not. Among those with a first birth <18, we compared those with and without repeat adolescent births (another birth <20 years). We used two-sample test for proportions, linear regression, and Poisson regression.

Findings

Among the 2,814 women age 40-49 analyzed, 36.2% reported a first birth <18 years and 85.9% of these had a repeat adolescent birth. Compared to women with no birth <18 years, those with first birth <18 years were less likely to have completed primary education (16.3% versus 32.2%, p<0.001), more likely to be illiterate (55.0% versus 44.0%, p<0.001), to report challenges seeking healthcare (67.6% versus 61.8%, p=0.002), and had higher mean number of births by age 40 years (6.6 versus 5.3, p<0.001). Among women married at time of survey, those with birth <18 years had older husbands (p<0.001) who also had lower educational attainment (p<0.001). Educational attainment, household wealth score, total number of births, and under-five mortality among women with one adolescent birth were similar, and sometimes better, than among those with no birth <18 years.

Conclusions

For peer review only - http://bmjopen.bmj.com/site/about/guidelines.xhtml

Results suggest life-long adverse socioeconomic and reproductive outcomes among women with adolescent birth, primarily in the category with repeat adolescent birth. While our results might be birth-cohort specific, they underscore the need to support adolescent mothers to have the same possibilities to develop their potentials, by supporting school continuation and prevention of further unwanted pregnancies.

Strengths and limitations of this study

- We used a nationally-representative sample of women in Uganda and assessed the extent of differential recall in reporting of adolescent births (which was minimal).
- The cross-sectional nature of the data and our descriptive analysis did not allow for assessment of causality.
- The results may largely apply to this cohort due to societal changes and advancements in protection of women but, this is a critical starting point to examine long term impact of early birth and repeat adolescent birth.

Funding: This work was supported through the Developing Excellence in Leadership, Training and Science (DELTAS) Africa Initiative grant # DEL-15-011 to THRiVE-2. The DELTAS Africa Initiative is an independent funding scheme of the African Academy of Sciences (AAS)'s Alliance for Accelerating Excellence in Science in Africa (AESA) and supported by the New Partnership for Africa's Development Planning and Coordinating Agency (NEPAD Agency) with funding from the Wellcome Trust grant # 107742/Z/15/Z and the UK government. The views expressed in this publication are those of the author(s) and not necessarily those of AAS, NEPAD Agency, Wellcome Trust or the UK government.

For peer review only - http://bmjopen.bmj.com/site/about/guidelines.xhtml

Competing interests: None declared.

Introduction

The sub-Saharan Africa region continues to have the worlds' highest burden of adolescent births, accounting for 95% of the 12 million births occurring to 15-19-year-old girls each year globally (1-5). Just as in other parts of sub-Saharan Africa, the levels of adolescent childbearing in Uganda remain unacceptably high: 24.8% of women aged 15-19 years have begun childbearing (pregnant or have given birth), levels similar to 15 years ago (6-9). According to the 2016 Uganda Demographic and Health Survey (DHS), 28.4% of women aged 20-24 years had an adolescent birth before age 18, a decline from 41.7% in 1988/89. Among women aged 20-24 years for whom the first birth occurred before age 18, the percentage reporting repeat adolescent birth (first birth <18, and another birth before the age of 20 years) did not decline in 30 years (58.9% in 1988/89, 55.6% in 2016) (10).

Adolescence (ages 10-19) is characterized by rapid physical, social, cognitive and emotional development that lays the foundation for an individual's health and wellbeing across the life course. As noted by Patton et al. (2018), adolescence is a period when individuals acquire health, social, cultural, financial and educational assets, whose effects can spill over to the next generation (11).

For peer review only - http://bmjopen.bmj.com/site/about/guidelines.xhtml

Global evidence shows that childbirth in adolescence, especially before the age of 18 years, is associated with poor social, economic and health outcomes for both the girl and her offspring (12-16). The health challenges associated with adolescent birth include obstructed labor, postpartum hemorrhage, preterm birth, fistulae, sepsis, and infant death among others (5, 12, 17). In addition, adolescent childbirth can negatively affect socio-economic outcomes leading to school dropout, limited cash income, and forced/early marriage (18-21). Married adolescents are more likely than those unmarried to initiate childbearing early, permanently drop out of school, and lack autonomy over their sexuality and reproduction due to patriarchal control (22-24). Younger adolescent girls are more prone to the negative outcomes due to their immature physiological state, poor economic independence, and developing cognition (25, 26). Having repeat pregnancies during adolescence can further exacerbate the risk of such negative outcomes (27, 28).

For peer review only

While the immediate effects are relatively described, there is limited evidence of the long-term socio-economic and reproductive health outcomes following early initiation of childbearing, including the effects of repeat adolescent birth, in Uganda and other low-income countries (29, 30). The available information, mainly from high-income countries, suggests life-long persistence of socio-economic disadvantage such as low educational attainment, poor earnings, and less stable marriages following an adolescent birth (31-34). A Population Council and Women Deliver report explored the short- and long-term socio-economic (employment and cash earnings) impact of having a child <18 years using the most recent DHS data sets from 43 LMICs including the 2016 Uganda DHS (34). This report suggested that economic disadvantages of adolescent childbirth persist over the entire lifetime for women. A study in Mexico found that women ages 25-64 years who had a birth between ages 15-19 years had lower educational attainment and lower income

For peer review only - http://bmjopen.bmj.com/site/about/guidelines.xhtml

compared to those who did not (33). Global literature points to persistence of the negative effects of adolescent birth on a woman's social welfare and household, including into the next generations (2, 5, 12, 35). A woman's agency and decision-making power can be negatively affected. For example, the resultant low educational and wealth attainment following early birth (5) is associated with high unmet need for contraception (36). In the long term, women who start childbirth during adolescence tend to end up with older partners who have lower educational and socio-economic attainment (32, 37, 38). However, these studies did not disaggregate outcomes among women who had *repeat* adolescent birth following the first birth <18 years, nor did they assess outcomes related to women's life-long reproductive health.

In response to this gap, this paper seeks to investigate the socio-economic and reproductive outcomes among women in Uganda toward the end of reproductive life-course (40-49 years) according to their adolescent birth history (no birth <18 years, first birth <18 years, repeat births <20 years). We hypothesize that having an adolescent birth before the age of 18 years leads to lifelong disruption in the acquisition of socio-economic resources and wellbeing. Further, we hypothesize that repeat adolescent births (first birth before the age of 18 and second before the of age 20 years) further worsens these outcomes. Given the link between early marriage and childbirth and its potential influence on later life outcomes (32, 39), we also stratified outcomes by marital status among women with first birth <18 years (comparing those with and without repeat adolescent birth).

For peer review only - http://bmjopen.bmj.com/site/about/guidelines.xhtml

Methods

Data sources and population

We used data from the 2016 Uganda DHS individual women's dataset. The DHS are nationally representative cross-sectional surveys where multi-stage cluster sampling is conducted. All the geographic regions of the country were represented. The DHS are conducted every five years in Uganda and collect information on population health along with socioeconomic and demographic characteristics. All women aged 15-49 years in sampled households for the individual women's dataset provided self-reported information about their live births. The interviewer-administered questionnaires used were translated into local languages and pre-tested prior to data collection.

The study population included women aged 40-49 years at the time of the 2016 survey, whom we categorized into three adolescent birth histories: 1) no birth <18 years; 2) first birth <18 years and no additional births <20 years; and 3) first birth <18 years and one or more additional births <20 years (repeat adolescent birth). We included live births rather than pregnancies and analyzed all women, including those who never gave birth, given that some pregnancies may end in miscarriage/abortion and others in stillbirths and therefore, the later life outcomes may not be impacted by this. To validate women's recall of adolescent childbearing, we conducted a sensitivity analysis comparing the childbirth patterns of women born in 1967-1976 as reported in the 1995 Uganda DHS to the same birth cohort of women as reported in 2016 (Supplementary file 1). We found similar estimates of first birth <18 years and repeat and no repeat adolescent births, indicating similar estimates of adolescent birth history among two representative samples of women from the same birth cohort in 1995 versus 2016.

Measures

For peer review only - http://bmjopen.bmj.com/site/about/guidelines.xhtml

Outcome variables: We assessed two main categories of later life outcomes: socio-economic and reproductive health-related. Supplementary file 2 contains the different variables assessed and their definitions. Socio-economic outcomes included: women's educational attainment (education level, means years of education); literacy; household wealth score (calculated using the household assets and presented as a linear index) (40); and receiving cash income at time of the 2016 Uganda DHS. Reproductive health outcomes included: number of live births by exact age 40 years; under-five mortality rate among any of the women's children; unmet need for contraception at the time of survey; and report of challenge seeking health care.

We further analyzed outcomes measured only in the sub-sample of women who were married or in a union at the time of the 2016 survey, including: age difference with husband; husband's education level (categorized in the same way as for women); and several measures of empowerment based on women's reports of whether they 1) solely decide on how their own earnings are spent, 2) are part of a marriage with other wives (polygamy), and 3) decide about their own health care.

Background variables included*:* women's area of residence (urban or rural); geographic region (Central, Eastern, Northern and Western); religion (Anglican, Catholic, Muslim, Other); marital status at survey (currently married/in union, divorced/separated, never in union); and the mean age (years) at survey, first sex, first birth, and at first union (if ever married/in union).

Statistical analysis

We began with exploratory data analysis to assess the distribution in outcomes and background characteristics and examine missingness. Next, we calculated the percentage of women according to their adolescent birth history (with vs. without first birth <18 years, and with vs. without repeat

For peer review only - http://bmjopen.bmj.com/site/about/guidelines.xhtml

adolescent birth). Column percentages of the later life outcomes and their 95% confidence intervals for each category of adolescent birth history were presented. We used the two-sample test of proportions to test for differences in the two column proportions for each later life outcome, and linear regression to compare group means for continuous variables. We further used Poisson regression to compare under-five mortality rates, estimating incidence risk ratios (IRR) and associated p-values. We described later life outcomes by adolescent birth history among all women and among the subsample of women who were married/in a union at time of survey (for whom partner information was available). We further described later life outcomes in a sub-analysis among women with and without repeat adolescent birth by marital status at first birth. For this sub-analysis, we included variables with p-value <0.05 between women with and without repeat adolescent births. Information on marital status at first birth were missing for N=95 (N=10 with no repeat adolescent birth and N=85 with repeat adolescent birth); these observations were therefore excluded from the sub-analysis.

We used survey weights, stratification and clustering to adjust for the complex survey design and non-response. All analyses were conducted using STATA version 12.0, StataCorp LP, Texas.

Patient and public involvement

For this study, we utilized secondary data and there was therefore no direct patient or public involvement. However, the study objectives and design were developed in collaboration with the Reproductive Health Division of the Ugandan Ministry of Health for whom adolescent fertility is a key research priority. The study builds on a previous landscape analysis of adolescent health needs conducted by the Ministry of Health, which indicated information gaps related to the long-

For peer review only - http://bmjopen.bmj.com/site/about/guidelines.xhtml

term effects of adolescent childbearing, especially for repeat adolescent birth. The results from the study will be disseminated to different stakeholders at the regional and national level.

Ethics

We obtained permission to use the datasets from the DHS program. During the survey data collection process, implemented by the Uganda Bureau of Statistics. written informed consent was obtained from all the respondents.

Results

From the analytical sample of 2,814 women aged 40-49 years in the 2016 Uganda DHS, 36.2% had their first birth <18 years (Figure 1). Among these, 85.9% reported a repeat adolescent birth (31.1% of all women). Similar proportions were observed for women who were married at the time of survey: 36.4% reported a first birth <18 years and of these, 87.1% reported a repeat adolescent birth (31.7% of all married women).

Table 1 shows the background characteristics of women in the 2016 sample based on their adolescent birth history. Most resided in rural areas (79.1%), belonged to the two main Christian religious faiths (Catholic and Anglican, 86.6%), and had ever been married or in a union (98%). The background characteristics were similar for women with and without first birth <18 years in terms of region, residence, religion, marital status, and mean age at survey. However, the mean age at first sex, first birth and first union differed by adolescent birth history. Women with first birth < age 18 reported earlier age at sexual debut (14.4 years vs. 17.3 years), and had their first birth and union earlier (15.5 and 16.3 years) compared to those who began childbearing after age

For peer review only - http://bmjopen.bmj.com/site/about/guidelines.xhtml

For peer review only

18. In contrast. women with repeat adolescent birth entered into marriage/union around the same age (15.9 years) as their first birth (15.5 years), whereas for those without repeat adolescent birth, first marriage or union (18.3 years) happened on average two years after their first birth (16.1 years). The average age at first birth for all women was 19.0 years whereas it was 15.5 years among those with first birth <18 years.

Later life outcomes by adolescent birth history

Looking at women all women aged 40-49 years (N=2814), results from bivariate analysis indicated that those who had their first birth <18 had significantly lower mean years of schooling (3.6 vs. 5.0 years, p<0.001), were less likely to only have completed primary school or higher (16.3% versus 32.2%, p<0.001), and more likely to be illiterate (55.0% vs 44.0%, p<0.001) compared to those without a birth <18 years (Table 2). Further, they also had lower household wealth score (p=0.043), were more likely to report health care seeking challenges (p=0.002), and had a higher mean number of live births by exact age 40 years (6.6 versus 5.3, p<0.001). There were no differences between the two groups in children's under-five mortality rate.

Among the 1,020 women with first birth <18 years, those with repeat adolescent birth were less likely than those without such experiences to have completed at least primary education (14.2% vs. 28.9%, p<0.001), to have lower mean years of education (3.4 vs. 4.7 years, p<0.001), household wealth scores (-0.003 vs. 0.020, p=0.032), more likely to be illiterate (57.1% vs. 41.9%, p<0.001), and to have higher mean number of live births by age 40 (7.0 versus 4.0, p<0.001). Further, the under-five mortality rate for children born to women with repeat adolescent births was higher than for those born to women with no repeat adolescent birth (IRR=1.36, 95% CI 1.06-

For peer review only - http://bmjopen.bmj.com/site/about/guidelines.xhtml

1.75). Health care seeking challenges and unmet need for contraceptives were, however, similar when comparing women with and without repeat adolescent births.

Analysis of a subsample of women 40-49 years married at time of 2016 survey

We further conducted analysis among a subsample of women who were married at time of the 2016 survey (N=2,022). Compared to women with no birth <18 years, those with first birth <18 years had partners/husbands who were older (7.6 versus 5.4 years older, p<0.001) and with lower education level (54.1% versus 61.2% primary school or above, p=0.002) (Table 3). Further, more women with first birth <18 years had control over how their own earnings are spent (p<0.001). There were no differences in outcomes regarding being in polygamous marriage and non-inclusion in decision-making about women's own healthcare. Among the 737 married women who had a first birth <18 years, there were no other differences in outcomes observed by whether they had repeat adolescent births.

Analysis of women with and without repeat adolescent births, by marital status at first birth

We also conducted analysis of later life outcomes among women with and without repeat adolescent birth by their marital status at first birth, including variables that were significantly associated with repeat adolescent birth (p<0.05) among all women aged 40-49 years (Table 2). The proportion married at first birth was higher for those with vs. without repeat adolescent birth (76.4% vs. 63.4%, p=0.001) (Table 4). Results showed no differences in later life outcomes based on marital status among women without repeat adolescent births. However, among women with repeat adolescent birth, those who were married at first birth had lower mean years of schooling

For peer review only

For peer review only - http://bmjopen.bmj.com/site/about/guidelines.xhtml

(3.2 vs. 3.9 years, p=0.017) and were more likely to be illiterate (59.7% vs. 49.3%, p=0.012) compared to those who were not married at the time.

Discussion

We investigated the socio-economic and reproductive health outcomes among Ugandan women towards the end of their reproductive life using data from the most recent Uganda DHS collected in 2016. We compared women's outcomes according to their adolescent birth histories: those with and without first birth <18 years, and among those with first birth <18 years, those with and without repeat adolescent births (two or more births before age 20). For the latter groups, we also examined differences in later life outcomes by marital status at the point of the first adolescent birth.

Our findings highlight three key points. First, women who had their first birth <18 years reported more negative socio-economic and reproductive health outcomes in later life than those without such experiences. These differences appear to be driven by the largest subgroup of women with repeat adolescent birth, who had poorer educational attainment, lower household wealth and empowerment, as well as higher fertility and under-five infant mortality compared to the other groups. This finding aligns with previous research highlighting adolescent childbirth as both a risk-factor for, and consequence of, poverty and low education (5, 41, 42). School discontinuation due to pregnancy is a persistent problem (42), and once a girl has given birth, she needs social, psychosocial and economic support to go back to school and complete her secondary education (5). Our findings further suggest that the risk of school discontinuation was greatest for girls who were married at first birth and went on to have repeat adolescent births, compared to their unmarried counterparts. It is possible that married adolescents with repeat adolescent birth are

For peer review only

For peer review only - http://bmjopen.bmj.com/site/about/guidelines.xhtml

taken out of school entirely (34, 42), underscoring the need to prevent early marriage in a country where marriage before age 18 remains common (40%) (43, 44), and thus a risk factor for repeat adolescent birth and its negative later life outcomes.

Secondly, women with an adolescent birth, and especially with repeat adolescent birth, were more likely to be socio-economically disadvantaged in later life – confirming the limited evidence on poor economic trajectories following early childbearing (33). For example, the Population Council and Women Deliver analysis of data from 43 low- and middle-income countries found that adolescents who gave birth before age 18 were significantly less likely to be earning cash, although there were no differences in employment (45). This suggests that low educational attainment and the burden of caring for many children ultimately lowered women's agency and chances of economic empowerment. This position is further supported by our finding of similar, or even better, socioeconomic outcomes among women with one adolescent birth compared to those with no birth <18 years; the lower mean number of children provided avenues for these women to pursue economic activities. Having a partner with a low educational attainment, as was the case for those with repeat adolescent births, may further have worsened the negative economic cycle. Indeed, women's opportunity for economic empowerment are not only linked to their educational attainment and literacy, but to the status of the spouse, which in turn impact household wealth scores (34, 46, 47). Our findings thus confirm the well-demonstrated link between poverty, low education and high fertility globally (5), calling for renewed efforts to alleviate household poverty and maintain adolescent girls in school.

We also found that women with first birth <18 years were more likely to report greater decision-making power over their own earnings, underscoring the results from the Population Council and Women Deliver report which found that adolescent birth was associated with more

For peer review only - http://bmjopen.bmj.com/site/about/guidelines.xhtml

control over own economic assets. This suggests that women with an adolescent birth might learn to be self-reliant and independent, providing a potential avenue for interventions to economically empower these women.

Third, our study results suggest that having a large completed family size is driven not only by an early initiation of childbearing, but by repeat adolescent births. Although the adolescent fertility rate has declined in Uganda, it remains high at 132 births per 1,000 women aged 15-19 years (9). This is reflected in how cultural norms in Uganda promote early marriage and childbearing, despite the legislation against early marriage (39, 42, 48, 49). The household poverty makes it worse, as girls are forced to discontinue schooling and get sent off into union in exchange for bride price (44, 50, 51). Low access and high unmet need for contraceptives among adolescent girls and young women in Uganda further restrict girls' reproductive agency in a heavily patriarchal context (52). Even in situations where an adolescent girl may want to use contraception, the partner or health worker may object to allow her to access the method (53). Further, abortion in Uganda is restricted to particular medical conditions, forcing girls facing an unintended pregnancy to either give birth or risk unsafe abortion (54, 55).

Strength and limitations

This study utilized data from a large nationally representative sample containing a variety of variables. However, the cross-sectional data cannot support causal associations between adolescent childbirth and the outcomes examined. While our assessment of differential reporting of adolescent childbirth did not show variation on a population level, the results may be influenced by reporting and recall bias due to the self-reported data. Women may also have given socially desirable responses regarding the age at first birth (56). In addition, some risk factors may have

For peer review only - http://bmjopen.bmj.com/site/about/guidelines.xhtml

been present right from early childhood. Due to this reverse causality, we cannot be certain of what came first; the negative outcomes or the birth. Nonetheless, this nationally representative survey data is a starting point to investigate later life outcomes in the absence of a prospective cohort in LMIC setting. Additionally, circumstances and opportunities available to women approximately 20 years ago may have altered and therefore, it may not fully depict the current situation in Uganda.

Conclusion

Findings from this study, while not necessarily causal, suggest that among women in Uganda, giving birth before age 18 is linked to lower educational attainment, household wealth, and higher fertility and under-five infant mortality outcomes later in life. Further, they tend to have spouses who are older and of low socio-economic attainment. This pattern is especially strong for women who have repeat adolescent births, and particularly for those who were married at first birth. Women who begin childbearing before age 18, but do not proceed to have a repeat adolescent birth – despite being a minority (5%) – appear to have recovered from any negative effects, and in some instances had better outcomes than those with no birth <18 years.

Public health interventions are needed to prevent repeat adolescent birth and potential negative life-course outcomes via improved access to continued schooling and higher education, and effective contraceptive services. Development of a Uganda school continuation policy for adolescent mothers needs to be fast tracked followed by: wide dissemination, sensitization of the communities and parents, and enforcement at school level (49-52, 57). Contraception information and services need to be made more accessible to all adolescents in a manner that promotes their autonomy. Further, adolescent women should be protected from early marriage by keeping them

For peer review only

For peer review only - http://bmjopen.bmj.com/site/about/guidelines.xhtml

in school and strengthening the legislation against early marriage (48, 50, 58). Our findings call for more in-depth research to explore and define what women without repeat adolescent birth did that enabled them to recuperate from the effects of early initiation of birth. Further, qualitative studies are needed to explore motivators and circumstances for repeat adolescent birth in sub-Saharan Africa. Finally, we suggest that secondary level completion among adolescent mothers be investigated further among younger cohorts, especially now that the Ministry of Education in Uganda, and many other countries in sub-Sahara Africa, permit girls to continue/resume schooling following pregnancy and childbirth (41).

The care of a child born to an adolescent girl takes broadly two patterns in Uganda and this cultural position needs to be factored in when viewing outcomes of women with early birth; those in and out of union. When a woman gives birth within marriage, the responsibility of raising the child is usually taken on by the man or his family with the support of the woman (59). In contrast, when they conceive outside union, the woman and child is usually sent off by the family to live with the man responsible for the pregnancy (18, 60, 61). In this situation, the man is assumed to take on the responsibility of raising the child. These cultural norms have remained essentially unchanged over the last four decades (50) despite advocacy for more paternal involvement in the care of children, most especially in scenarios of no union/marriage. Women out of union would most probably have been left with the entire burden of the offspring(s).

For peer review only

References:

1. Temmerman M. Adolescent mothers: too young to be neglected. The Lancet Child & Adolescent Health. 2017;1(3):164-6.

For peer review only - http://bmjopen.bmj.com/site/about/guidelines.xhtml

2. Timæus IM, Moultrie TA. Teenage Childbearing and Educational Attainment in South Africa. Studies in Family Planning. 2015;46(2):143-60.
3. Neal S, Matthews Z, Frost M, Fogstad H, Camacho AV, Laski L. Childbearing in adolescents aged 12-15 years in low resource countries: a neglected issue. New estimates from demographic and household surveys in 42 countries. Acta Obstet Gynecol Scand. 2012;91(9):1114-8.
4. Yakubu I, Salisu WJ. Determinants of adolescent pregnancy in sub-Saharan Africa: a systematic review. Reproductive health. 2018;15(1):15.
5. WHO. Adolescent pregnancy. Fact sheet. World Health Organization; 2020 31 January 2020.
6. Neal S, Ruktanonchai C, Chandra-Mouli V, Matthews Z, Tatem AJ. Mapping adolescent first births within three east African countries using data from Demographic and Health Surveys: exploring geospatial methods to inform policy. Reprod Health. 2016;13(1):98.
7. Neal SE, Chandra-Mouli V, Chou D. Adolescent first births in East Africa: disaggregating characteristics, trends and determinants. Reproductive health. 2015;12(1):13.
8. Wado YD, Sully EA, Mumah JN. Pregnancy and early motherhood among adolescents in five East African countries: a multi-level analysis of risk and protective factors. BMC pregnancy and childbirth. 2019;19(1):59.
9. UBOS. Uganda Demographic and Health Survey 2016. Kampala, Uganda and Rockville, Maryland, USA: UBOS and ICF; 2018 2018.
10. Amongin D, Nakimuli A, Hanson C. Time trends in and factors associated with repeat adolescent birth in Uganda: Analysis of six demographic and health surveys. 2020;15(4):e0231557.
11. Patton GC, Olsson CA, Skirbekk V, Saffery R, Wlodek ME, Azzopardi PS, et al. Adolescence and the next generation. Nature. 2018;554(7693):458-66.
12. Loredo-Abdala A, Vargas-Campuzano E, Casas-Munoz A, Gonzalez-Corona J, Gutierrez-Leyva CJ. [Adolescent pregnancy: its causes and repercussions in the dyad]. Rev Med Inst Mex Seguro Soc. 2017;55(2):223-9.
13. Pérez MM, Barquero ML, Sánchez ML, De L, Los Rios Gestoso GTR, Aso CM, et al. CONSEQUENCES OF TEEN PREGNANCY.
14. Pinto ESJL, Surita FG. Pregnancy in Adolescence - A Challenge Beyond Public Health Policies. Rev Bras Ginecol Obstet. 2017;39(2):41-3.
15. Blanc AK, Winfrey W, Ross J. New findings for maternal mortality age patterns: aggregated results for 38 countries. PloS one. 2013;8(4):e59864.
16. Nove A, Matthews Z, Neal S, Camacho AV. Maternal mortality in adolescents compared with women of other ages: evidence from 144 countries. The Lancet Global Health. 2014;2(3):e155-e64.
17. United Nations DoEaSA, Population Division. World Fertility 2019: Early and later childbearing among adolescent women. 2020.
18. Atuyambe L, Mirembe F, Johansson A, Kirumira EK, Faxelid E. Experiences of pregnant adolescents--voices from Wakiso district, Uganda. Afr Health Sci. 2005;5(4):304-9.
19. Mollborn S. Teenage Mothers Today: What We Know and How It Matters. Child Development Perspectives. 2017;11(1):63-9.
20. Diaz CJ, Fiel JE. The effect (s) of teen pregnancy: Reconciling theory, methods, and findings. Demography. 2016;53(1):85-116.
21. Ranchhod V, Lam D, Leibbrandt M, Marteleto L. Estimating the effect of adolescent fertility on educational attainment in Cape Town using a propensity score weighted regression. 2011.
22. Adesina M, Daha M, Ajobiewe A, Oladele RI, Olufadewa I, Ayorinde T. The High Proportion of Child Brides in Africa: Implications for The Girl Child and The Society. 2020.

For peer review only - http://bmjopen.bmj.com/site/about/guidelines.xhtml

23. Sunmola AK, Olaosebikan JS, Adeusi TJ. High fertility level in Sub-Saharan Africa: implication for reaping and optimizing demographic dividend. International Journal of Community Medicine and Public Health. 2020;7(9):3698.

24. Delprato M, Akyeampong K, Dunne M. Intergenerational education effects of early marriage in Sub-Saharan Africa. World Development. 2017;91:173-92.

25. Leftwich HK, Alves MVO. Adolescent Pregnancy. Pediatric Clinics of North America. 2017;64(2):381-8.

26. Chen X-K, Wen SW, Fleming N, Demissie K, Rhoads GG, Walker M. Teenage pregnancy and adverse birth outcomes: a large population based retrospective cohort study. International journal of epidemiology. 2007;36(2):368-73.

27. Maravilla JC, Betts KS, Alati R. Increased risk of maternal complications from repeat pregnancy among adolescent women. International Journal of Gynecology & Obstetrics. 2019.

28. Maravilla JC, Betts KS, Alati R. Trends in repeated pregnancy among adolescents in the Philippines from 1993 to 2013. Reproductive health. 2018;15(1):184.

29. Fall CH, Sachdev HS, Osmond C, Restrepo-Mendez MC, Victora C, Martorell R, et al. Association between maternal age at childbirth and child and adult outcomes in the offspring: a prospective study in five low-income and middle-income countries (COHORTS collaboration). The Lancet Global Health. 2015;3(7):e366-e77.

30. Ellis-Sloan K. Teenage mothers in later life: Time for a second look. Journal of Adolescence. 2019;77:98-107.

31. Lounds Taylor J. Midlife impacts of adolescent parenthood. Journal of family issues. 2009;30(4):484-510.

32. Hoffman SD, Maynard RA. Kids having kids: Economic costs & social consequences of teen pregnancy: The Urban Insitute; 2008.

33. Arceo-Gómez EO, Campos-Vázquez RM. Teenage pregnancy in Mexico: evolution and consequences. Latin american journal of economics. 2014;51(1):109-46.

34. Women Deliver and The Population Council. Having a child before becoming an adult. Exploring the economic impact in a multi-country Analysis. Report. New York: Women Deliver; 2019 2019.

35. Aitken Z, Hewitt B, Keogh L, LaMontagne AD, Bentley R, Kavanagh AM. Young maternal age at first birth and mental health later in life: Does the association vary by birth cohort? Social Science & Medicine. 2016;157:9-17.

36. Ethiopia K. What it takes: Meeting unmet need for family planning in East Africa. Policy. 2018.

37. Card JJ, Wise LL. Teenage mothers and teenage fathers: The impact of early childbearing on the parents' personal and professional lives. Family planning perspectives. 1978;10(4):199-205.

38. Ermisch J. Does a'teen-birth'have longer-term impacts on the mother? suggestive evidence from the British Household Panel Study. ISER Working Paper Series; 2003.

39. Walker J-A. Early marriage in Africa–trends, harmful effects and interventions. African journal of reproductive health. 2012;16(2):231-40.

40. Filmer D, Pritchett LH. Estimating wealth effects without expenditure data—or tears: an application to educational enrollments in states of India. Demography. 2001;38(1):115-32.

41. Watch HR. LEAVE NO GIRL BEHIND IN AFRICA: Discrimination in Education against Pregnant Girls and Adolescent Mothers. 2018.

42. Wodon Q, Nguyen MC, Tsimpo C. Child marriage, education, and agency in Uganda. Feminist Economics. 2016;22(1):54-79.

43. Male C, Wodon Q. Basic profile of child marriage in Uganda. 2016.

44. Petroni S, Steinhaus M, Fenn NS, Stoebenau K, Gregowski A. New findings on child marriage in sub-Saharan Africa. Annals of global health. 2017;83(5-6):781-90.

For peer review only - http://bmjopen.bmj.com/site/about/guidelines.xhtml

45. Wilkie GL, Leung K, Kumaraswami T, Barlow E, Simas TAM. Effects of Obstetric Complications on Adolescent Postpartum Contraception and Rapid Repeat Pregnancy. Journal of pediatric and adolescent gynecology. 2016;29(6):612-6.
46. Rasmussen B, Maharaj N, Sheehan P, Friedman HS. Evaluating the employment benefits of education and targeted interventions to reduce child marriage. Journal of Adolescent Health. 2019;65(1):S16-S24.
47. Kanjilal-Bhaduri S, Pastore F. Returns to education and female participation nexus: Evidence from India. The Indian Journal of Labour Economics. 2018;61(3):515-36.
48. Addaney M, Azubike O-O. Education as a Contrivance to Ending Child Marriage in Africa: Perspective from Nigeria and Uganda. Amsterdam LF. 2017;9:110.
49. Leerlooijer JN, Bos AE, Ruiter RA, Van Reeuwijk MA, Rijsdijk LE, Nshakira N, et al. Qualitative evaluation of the Teenage Mothers Project in Uganda: a community-based empowerment intervention for unmarried teenage mothers. BMC public health. 2013;13(1):816.
50. Bantebya GK, Muhanguzi FK, Watson C. Adolescent girls in the balance: Changes and continuity in social norms and practices around marriage and education in Uganda. Overseas Development Institute: London, UK. 2014.
51. Okwany A, Kamusiime A. Foregrounding the tensions and silences in education policies for student-mothers in Uganda and Kenya. Childhood in Africa. 2017;4(1).
52. Wodon QT, Male C, Onagoruwa AO, Savadogo A, Yedan A. The cost of not investing in girls: child marriage, early childbearing, low educational attainment for girls, and their impacts in Uganda. The World Bank; 2017.
53. Nalwadda G, Mirembe F, Byamugisha J, Faxelid E. Persistent high fertility in Uganda: young people recount obstacles and enabling factors to use of contraceptives. BMC public health. 2010;10(1):530.
54. Sully EA, Atuyambe L, Bukenya J, Whitehead HS, Blades N, Bankole A. Estimating abortion incidence among adolescents and differences in postabortion care by age: a cross-sectional study of postabortion care patients in Uganda. Contraception. 2018;98(6):510-6.
55. Atuyambe LM, Kibira SP, Bukenya J, Muhumuza C, Apolot RR, Mulogo E. Understanding sexual and reproductive health needs of adolescents: evidence from a formative evaluation in Wakiso district, Uganda. Reproductive health. 2015;12(1):1-10.
56. Neal SE, Hosegood V. How Reliable Are Reports of Early Adolescent Reproductive and Sexual Health Events in Demographic and Health Surveys? Int Perspect Sex Reprod Health. 2015;41(4):210-7.
57. Birungi H, Undie C-C, MacKenzie I, Katahoire A, Obare F, Machawira P. Education sector response to early and unintended pregnancy: A review of country experiences in Sub-Saharan Africa. 2015.
58. Kalamar AM, Lee-Rife S, Hindin MJ. Interventions to prevent child marriage among young people in low-and middle-income countries: a systematic review of the published and gray literature. Journal of Adolescent Health. 2016;59(3):S16-S21.
59. Ickes SB, Heymsfield GA, Wright TW, Baguma C. “Generally the young mom suffers much:” Socio-cultural influences of maternal capabilities and nutrition care in Uganda. Maternal & child nutrition. 2017;13(3):e12365.
60. Ninsiima AB, Leye E, Michielsen K, Kemigisha E, Nyakato VN, Coene G. “Girls Have More Challenges; They Need to Be Locked Up”: A Qualitative Study of Gender Norms and the Sexuality of Young Adolescents in Uganda. International journal of environmental research and public health. 2018;15(2):193.
61. Kaye DK. Negotiating the transition from adolescence to motherhood: Coping with prenatal and parenting stress in teenage mothers in Mulago hospital, Uganda. BMC Public Health. 2008;8(1):1-6.

For peer review only

For peer review only - http://bmjopen.bmj.com/site/about/guidelines.xhtml

Table 1: Background characteristics of Uganda women 40-49 years by adolescent birth history; 2016 Uganda DHS

	All women 40-49 years *col% (95% CI)*	No birth <18 years *col% (95% CI)*	1st Birth <18 years *col% (95% CI)*	Birth <18 years, no repeat *col% (95% CI)*	Birth <18 years, Repeat *col% (95% CI)*
**Variables**	**N=2,814**	**N=1,795**	**N=1,020**	**N=144**	**N=876**
**Residence**					
Urban	20.9 (18.7-23.3)	21.0 (18.3-23.9)	20.8 (17.7-24.3)	23.7 (16.4-33.1)	20.3 (17.4-23.7)
Rural	79.1 (76.7-81.3)	79.0 (76.1-81.7)	79.2 (75.7-82.3)	76.3 (66.9-83.6)	79.7 (76.3-82.6)
**Region**					
Central	26.5 (22.3-31.1)	24.4) (20.2-29.3	30.1 (24.8-36.0)	31.4 (21.8-42.9)	29.9 (24.3-36.1)
Eastern	30.3 (26.1-34.9)	29.4 (25.1-34.1)	31.9 (26.8-37.6)	30.2 (21.4-40.6)	32.2 (26.9-38.1)
Northern	17.6 (14.6-21.1)	18.3 (15.1-22.1)	16.5 (13.2-20.2)	18.1 (12.3-26.0)	16.2 (12.9-20.0)
Western	25.6 (21.7-29.8)	27.9 (23.5-32.7)	21.5 (17.6-26.0)	20.3 (13.8-28.9)	21.7 (17.6-26.4)
**Religion**					
Anglican	44.6 (42.0-47.2)	44.8 (41.9-47.9)	44.1 (40.1-48.1)	39.4 (30.6-48.9)	44.8 (40.6-49.2)
Catholic	42.0 (39.2-44.9)	43.2 (39.9-46.5)	40.0 (36.2-43.9)	48.0 (38.9-57.2)	38.7 (34.6-43.0)
Muslim	10.5 (8.8-12.6)	9.1 (7.2-11.4)	13.0 (10.4-16.2)	9.2 (5.3-15.7)	13.7 (10.8-17.1)
Other	2.9 (2.2-3.8)	2.9 (2.1-4.0)	2.9 (1.8-4.5)	3.4 (1.2-9.2)	2.8 (1.7-4.5)
**Marital status at survey**					
Never in union	2.0 (1.5-2.7)	2.4 (1.7-3.4)	1.3 (0.7-2.3)	0.3 (0.0-2.0)	1.4 (0.8-2.6)
Currently in union/marriage	71.8 (69.9-73.7)	71.6 (69.1-73.9)	72.3 (69.0-75.4)	66.4 (57.1-74.5)	73.3 (69.8-76.5)
Formerly in union/marriage	26.2 (24.3-28.1)	26.0 (23.7-28.5)	26.4 (23.4-29.7)	33.4 (25.2-42.6)	25.3 (22.1-28.7)
**Mean age at survey (sd)**	43.9 (2.9)	44.1 (2.8)	43.8 (2.9)	43.5 (2.9)	43.9 (2.9)
**Mean age at first sex (sd)**	16.3 (2.8)	17.3 (2.8)	14.4 (1.5)	15.1 (1.4)	14.3 (1.5)
**Mean age at 1st birth (sd)**	19.0 (3.9)	21.1 (3.3)	15.5 (1.5)	16.1 (1.2)	15.5 (1.5)
**Mean age at 1st union, if ever married (SD)**	18.9 (5.3)	20.3 (4.9)	16.3 (5.0)	18.3 (5.8)	15.9 (4.8)

For peer review only

For peer review only - http://bmjopen.bmj.com/site/about/guidelines.xhtml

Table 2: Socioeconomic and health-related life course outcomes by adolescent birth history among Uganda women 40-49 years, 2016 Uganda DHS (N=2815)

	**All women 40-49 years**			**Women with first birth <18 years**		
	No birth <18 years **(N=1795)**	Birth <18 years **(N=1020)**		No repeat adolescent birth **(N=144)**	Repeat adolescent birth **(N=876)**	
**Variables**	% (95% CI)	% (95% CI)	***p***	% (95% CI)	% (95% CI)	*p*
**Education level**						
Complete primary and above	32.2 (29.3-35.3)	16.3 (13.7-19.2)	<0.001	28.9 (21.2-37.9)	14.2 (11.6-17.3)	<0.001
**Mean years in school (SD)**	5.0 (4.4)	3.6 (3.2)	<0.001	4.7 (3.4)	3.4 (3.1)	0.001
**Literacy**						
Illiterate (cannot read at all)	44.0 (40.9-47.1)	55.0 (50.8-59.1)	<0.001	41.9 (32.7-51.8)	57.1 (52.8-61.4)	<0.001
**Household wealth score**	0.009 (0.100)	0.001 (0.085)	0.043	0.020 (0.099)	-0.003 (0.082)	0.032
**Receiving cash income at survey (yes)**	70.0 (67.0-72.8)	70.5 (67.1-73.7)	0.780	71.3 (61.7-79.2)	70.4 (66.7-73.8)	0.826
**Challenge seeking health care (yes)**	61.8 (58.6-64.9)	67.6 (63.9-71.1)	0.002	62.0 (52.2-70.9)	68.5 (64.5-72.3)	0.123
**Mean No. of live births by exact age 40 years (SD)**	5.3 (2.1)	6.6 (1.7)	<0.001	4.0 (1.6)	7.0 (1.3)	<0.001
**U5 mortality rate**	124.6	140.1	P=0.341	104.8	142.9	P=0.016
**Unmet need for contraception**	19.8 [17.8,21.9]	20.2 (17.4-23.4)	0.799	20.1 (13.0-29.6)	20.2 [17.1,23.8]	0.978

* Under5mortality: Divided by 1000 live births. P-value obtained for the IRR that compared the mortality rate between 2 groups. P=0.016, IRR was 1.36 (95% CI 1.06-0.75).

For peer review only

For peer review only - http://bmjopen.bmj.com/site/about/guidelines.xhtml

Table 3: Later life outcomes related to partner characteristics among a subsample of married women 40-49 (2016 Uganda DHS, N=2022)

	All women 40-49 years			Women with first birth <18 years		
	**No birth <18 years (N=1285)**	**Birth <18 years (N=737)**		**No repeat adol. birth (N=096)**	**Repeat adol. birth (N=642)**	
**Variables**	% (95% CI)	% (95% CI)	*p*	% (95% CI)	% (95% CI)	*p*
**Mean age difference with partner/husband- years (SD)**	5.4 (6.4)	7.6 (7.2)	<0.001	6.8 (7.1)	7.7 (7.2)	0.358
**Husband's education level**	N=1285	N=737		N=96	N=642	
Primary	54.1 (50.5-57.7)	61.2 (56.9-65.3)	0.002	60.0 (48.6-70.5)	61.3 (56.8-65.6)	0.808
Secondary/higher	34.1 (30.7-37.7)	26.6 (22.9-30.6)	<0.001	31.7 (22.2-43.0)	25.8 (22.0-30.0)	0.222
**Women who solely decide on how her earnings are spent**	N=897<br>52.1 (48.3-55.9)	N=499<br>64.2 (58.8-69.3)	<0.001	N=68<br>64.8 (50.2-77.0)	N=499<br>64.1 (58.6-69.3)	0.910
**Women not included on decisions about:**						
Their own health care	18.8 (16.4-21.4)	20.9 (17.5-24.8)	0.252	15.5 (8.7-26.0)	21.8 (17.9-26.2)	0.157
**In marriage with other wives (polygamy)**	33.1 (29.8-36.6)	36.2 (32.3-40.2)	0.157	42.9 (31.1-55.6)	35.2 (31.1-39.4)	0.143

For peer review only

For peer review only - http://bmjopen.bmj.com/site/about/guidelines.xhtml

Table 4: Later life outcomes among women 40-49 years with and without repeat adolescent birth, by marital status at first birth (N=925)

	**No repeat adolescent birth**			**Repeat adolescent birth**		
	**Not married at 1st birth (N=49)**	**Married at 1st birth (N=85)**		**Not married at 1st birth (N=186)**	**Married at 1st birth (N=604)**	
**Variables**	% (95% CI)	% (95% CI)	***p***	% (95% CI)	% (95% CI)	***p***
**Education level**						
Complete primary and above	29.6 (17.5-45.4)	24.7 (15.7-36.5)	0.536	17.4 (12.1-24.4)	12.4 (9.7-15.8)	0.082
**Mean years in school (SD)**	4.7 (3.0)	4.2 (3.2)	0.458	3.9 (3.0)	3.2 (3.1)	0.017
**Literacy**						
Cannot read at all (illiterate)	40.3 (25.2-57.5)	44.4 (32.2-57.4)	0.644	49.3 (40.1-58.6)	59.7 (54.7-64.5)	0.012
**Household wealth scores** (SD)	0.005 (0.082)	0.024 (0.097)	0.320	0.004(0.085)	-0.008 (0.078)	0.097
**Mean No. of live births by exact age 40 years** (39.99) (SD)	4.0 (1.7)	4.2 (1.6)	0.602	7.0 (1.2)	7.0 (1.3)	0.963
**U5 mortality rate**/1000 live births	109.3	107.1	0.884	140.4	144.6	0.803

NB: * P-value of the IRR obtained from comparing the mortality rates. P=0.884 (IRR 0.98, 95%CI=0.75-1.28) and p=0.803 (IRR 1.03, 95% CI=0.82-1.30)

Abbreviations: BMI, Body Mass Index; DHS, Demographic and Health Surveys; FP, Family Planning; IRR, Incidence risk Ratio; LMIC, Low-and-Middle-Income Country; SD, Standard Deviation; STI, Sexually Transmitted Infection; U5, Under-five.

Author contributions:

DA conceived and designed the study, obtained permission to use data set from DHS program, conducted analysis, presented and interpreted results, drafted and finalized the article. AK conceived the study, conducted the literature review, reviewed and interpreted the results, and reviewed the article. ÖT conceived the study, drafted the analysis plan, reviewed and interpreted the results and reviewed the article. AN was involved reviewing the study design, the results and drafting the article. MN was involved in data analysis, presentation and interpretation of the results.

For peer review only

For peer review only - http://bmjopen.bmj.com/site/about/guidelines.xhtml

LA was involved in reviewing and interpreting the results and reviewing the manuscript. CH was involved in designing the study, the analysis, presenting and interpreting the results, drafting the article and reviewing the manuscript. LB was involved in conceiving the study, designing the analysis plan, data analysis, interpreting the results and substantially reviewing the article. All the authors reviewed and approved the manuscript. All the authors take responsibility for their contributions. None of the authors has competing interests.

Data sharing: Data used for this analysis can be accessed, with permission, from the Demographic and Health Survey program website https://www.dhsprogram.com/data/available-datasets.cfm

For peer review only

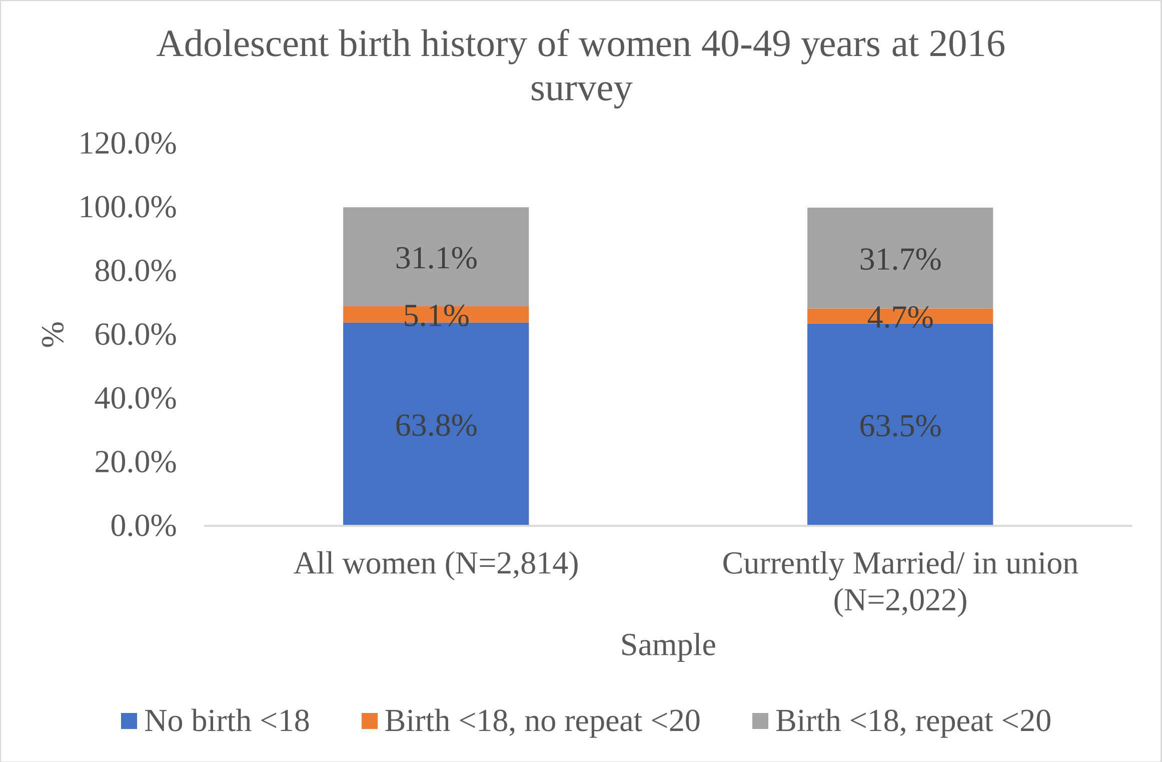

Figure 1: Adolescent birth history among Uganda women aged 40-49 years at the 2016 Uganda DHS

Supplementary table 1: Adolescent birth history among women born between 1967-1976 (1995 and 2016 UDHS)

Adolescent fertility pattern	UDHS 1995	UDHS 2016
	N=2,723	N=2,841
	% (95% CI)	% (95% CI)
**No birth <18**	61.0 (58.6-63.5)	63.4 (61.2-65.5)
**Birth <18, no repeat birth <20**	15.0 (13.6-16.5)	14.0 (12.6-15.5)
**Birth <18, repeat birth <20**	24.0 (22.0-26.1)	22.7 (20.9-24.6)

For peer review only

Supplementary table 2: The variable definitions/categorization for the analysis of the 2016 UDHS

Variable	Categories/definitions
**Residence**	• Rural<br>• Urban
**Region**	• Central (Kampala, Central 1 (South Buganda) & Central 2 (North Buganda))<br>• Eastern (Teso, Karamoja, Bugisu, Bukedi, & Busoga sub-regions)<br>• Northern (Lango, Acholi & West Nile sub-regions)<br>• Western (Bunyoro, Tooro, Ankole & Kigezi sub-regions)
**Religion**	• Anglican (Anglican & Pentecostal/born again/evangelical)<br>• Catholic<br>• Muslim<br>• Other (Seventh day Adventist, orthodox, Baptist, traditional, no religion, other, & Jehovah's Witness)
**Marital status at survey**	• Never in union<br>• Currently in union/marriage (married and living with partner)<br>• Formerly in union/marriage (widowed, divorced, and no longer living together/separated).
**Socioeconomic**	
**Educational attainment**	
Education level	• Incomplete primary (no education & incomplete primary)<br>• Complete primary and higher (Complete primary, incomplete secondary, complete secondary, and higher)
Mean number of years in school (education)	Maintained as a continuous variable
**Literacy**	• cannot read at all (illiterate)<br>• can read (able to read only parts of sentence & able to read whole sentence)<br>• no card with required language<br>• blind/visually impaired
**Receiving cash income at survey**	• Yes (cash only & cash and in-kind)<br>• No (not paid, in-kind only, & missing)

For peer review only

STROBE Statement—Checklist of items that should be included in reports of ***cross-sectional studies***

	Item No	Recommendation	Page No
**Title and abstract**	1	(*a*) Indicate the study's design with a commonly used term in the title or the abstract	1
		(*b*) Provide in the abstract an informative and balanced summary of what was done and what was found	3
**Introduction**			
Background/rationale	2	Explain the scientific background and rationale for the investigation being reported	6
Objectives	3	State specific objectives, including any prespecified hypotheses	7
**Methods**			
Study design	4	Present key elements of study design early in the paper	8
Setting	5	Describe the setting, locations, and relevant dates, including periods of recruitment, exposure, follow-up, and data collection	8
Participants	6	(*a*) Give the eligibility criteria, and the sources and methods of selection of participants	8
Variables	7	Clearly define all outcomes, exposures, predictors, potential confounders, and effect modifiers. Give diagnostic criteria, if applicable	9
Data sources/ measurement	8*	For each variable of interest, give sources of data and details of methods of assessment (measurement). Describe comparability of assessment methods if there is more than one group	*9*
Bias	9	Describe any efforts to address potential sources of bias	10
Study size	10	Explain how the study size was arrived at	8
Quantitative variables	11	Explain how quantitative variables were handled in the analyses. If applicable, describe which groupings were chosen and why	9
Statistical methods	12	(*a*) Describe all statistical methods, including those used to control for confounding	10
		(*b*) Describe any methods used to examine subgroups and interactions	9
		(*c*) Explain how missing data were addressed	11
		(*d*) If applicable, describe analytical methods taking account of sampling strategy	11
		(*e*) Describe any sensitivity analyses	8
**Results**			
Participants	13*	(a) Report numbers of individuals at each stage of study—eg numbers potentially eligible, examined for eligibility, confirmed eligible, included in the study, completing follow-up, and analysed	12
		(b) Give reasons for non-participation at each stage	NA
		(c) Consider use of a flow diagram	NA
Descriptive data	14*	(a) Give characteristics of study participants (eg demographic, clinical, social) and information on exposures and potential confounders	12
		(b) Indicate number of participants with missing data for each variable of interest	11
Outcome data	15*	Report numbers of outcome events or summary measures	13
Main results	16	(*a*) Give unadjusted estimates and, if applicable, confounder-adjusted estimates and their precision (eg, 95% confidence interval). Make clear which confounders were adjusted for and why they were included	13

For peer review only

		(*b*) Report category boundaries when continuous variables were categorized	13
		(*c*) If relevant, consider translating estimates of relative risk into absolute risk for a meaningful time period	NA
Other analyses	17	Report other analyses done—eg analyses of subgroups and interactions, and sensitivity analyses	13/14
**Discussion**			
Key results	18	Summarise key results with reference to study objectives	16
Limitations	19	Discuss limitations of the study, taking into account sources of potential bias or imprecision. Discuss both direction and magnitude of any potential bias	19
Interpretation	20	Give a cautious overall interpretation of results considering objectives, limitations, multiplicity of analyses, results from similar studies, and other relevant evidence	20
Generalisability	21	Discuss the generalisability (external validity) of the study results	21
**Other information**			
Funding	22	Give the source of funding and the role of the funders for the present study and, if applicable, for the original study on which the present article is based	2

*Give information separately for exposed and unexposed groups.

Note: An Explanation and Elaboration article discusses each checklist item and gives methodological background and published examples of transparent reporting. The STROBE checklist is best used in conjunction with this article (freely available on the Web sites of PLoS Medicine at http://www.plosmedicine.org/, Annals of Internal Medicine at http://www.annals.org/, and Epidemiology at http://www.epidem.com/). Information on the STROBE Initiative is available at www.strobe-statement.org.

For peer review only

BMJ Open

Later Life Outcomes of Women by Adolescent Birth History: Analysis of the 2016 Uganda Demographic and Health Survey

Journal:	*BMJ Open*
Manuscript ID	bmjopen-2020-041545.R2
Article Type:	Original research
Date Submitted by the Author:	29-Jan-2021

Complete List of Authors:	Amongin, Dinah; Makerere University College of Health Sciences, Department of Health Policy Planning and Management<br>Kågesten, Anna; Karolinska Institutet, Department of Global Public Health<br>Tunçalp, Özge; World Health Organizations, 4. Department of Sexual and Reproductive Health and Research, including the UNDP/UNFPA/UNICEF/WHO/World Bank Special Programme of Research, Development and Research Training in Human Reproduction (HRP)<br>Nakimuli, A; Makerere University College of Health Sciences, Department of Obstetrics and Gynecology<br>Nakafeero, Mary; Makerere University School of Public Health, Department of Epidemiology and Biostatistics<br>Atuyambe, Lynn ; Makerere University School of Public Health, Department of Community Health and Behavioral Sciences<br>Hanson, Claudia; Karolinska Institute, Department of Global Public Health; London School of Hygiene and Tropical Medicine Faculty of Infectious and Tropical Diseases, Dept of Disease Control<br>Benova, Lenka; Institute of Tropical Medicine, Department of Public Health; London School of Hygiene & Tropical Medicine, Faculty of Epidemiology and Population Health

**Primary Subject Heading**:	Obstetrics and gynaecology
Secondary Subject Heading:	Epidemiology, Global health, Health policy, Obstetrics and gynaecology, Sexual health
Keywords:	OBSTETRICS, REPRODUCTIVE MEDICINE, PUBLIC HEALTH, SEXUAL MEDICINE

SCHOLARONE™
Manuscripts

I, the Submitting Author has the right to grant and does grant on behalf of all authors of the Work (as defined in the below author licence), an exclusive licence and/or a non-exclusive licence for contributions from authors who are: i) UK Crown employees; ii) where BMJ has agreed a CC-BY licence shall apply, and/or iii) in accordance with the terms applicable for US Federal Government officers or employees acting as part of their official duties; on a worldwide, perpetual, irrevocable, royalty-free basis to BMJ Publishing Group Ltd ("BMJ") its licensees and where the relevant Journal is co-owned by BMJ to the co-owners of the Journal, to publish the Work in this journal and any other BMJ products and to exploit all rights, as set out in our licence.

The Submitting Author accepts and understands that any supply made under these terms is made by BMJ to the Submitting Author unless you are acting as an employee on behalf of your employer or a postgraduate student of an affiliated institution which is paying any applicable article publishing charge ("APC") for Open Access articles. Where the Submitting Author wishes to make the Work available on an Open Access basis (and intends to pay the relevant APC), the terms of reuse of such Open Access shall be governed by a Creative Commons licence – details of these licences and which Creative Commons licence will apply to this Work are set out in our licence referred to above.

Other than as permitted in any relevant BMJ Author's Self Archiving Policies, I confirm this Work has not been accepted for publication elsewhere, is not being considered for publication elsewhere and does not duplicate material already published. I confirm all authors consent to publication of this Work and authorise the granting of this licence.

For peer review only

Later Life Outcomes of Women by Adolescent Birth History: Analysis of the 2016 Uganda Demographic and Health Survey

Dinah Amongin[1,2*], Anna E. Kågesten[3], Özge Tunçalp[4], Annettee Nakimuli[2], Mary Nakafeero[5], Lynn M. Atuyambe[6], Claudia Hanson [3,7], Lenka Benova[8,9]

1. Department of Health Policy Planning and Management, Makerere University School of Public Health
2. Department of Obstetrics and Gynaecology, School of Medicine, Makerere University College of Health Sciences
3. Department of Global Public Health, Karolinska Institutet, Sweden
4. Department of Sexual and Reproductive Health and Research, including the UNDP/UNFPA/UNICEF/WHO/World Bank Special Programme of Research, Development and Research Training in Human Reproduction (HRP), World Health Organization, Switzerland
5. Department of Epidemiology and Biostatistics, Makerere University School of Public Health
6. Department of Community Health and Behavioral Sciences, Makerere University School of Public Health
7. Department of Disease Control, London School of Hygiene & Tropical Medicine, UK
8. Faculty of Epidemiology and Population Health, London School of Hygiene and Tropical Medicine
9. Department of Public Health, Institute of Tropical Medicine, Antwerp Belgium

***Corresponding author:**

Email**:** amongdinah2003@yahoo.com

Tel: +256704270097

Fax: +256 414 531807

For peer review only - http://bmjopen.bmj.com/site/about/guidelines.xhtml

Abstract

Objectives: To describe the long-term socio-economic and reproductive health outcomes of women in Uganda by adolescent birth history.

Design: Cross-sectional study

Setting: Uganda

Participants: Women aged 40-49 years at the 2016 Uganda Demographic and Health Survey.

Outcome measures: We compared socioeconomic and reproductive outcomes among those with first birth <18 years versus not. Among those with a first birth <18, we compared those with and without repeat adolescent births (another birth <20 years). We used two-sample test for proportions, linear regression, and Poisson regression.

Findings

Among the 2,814 women age 40-49 analyzed, 36.2% reported a first birth <18 years and 85.9% of these had a repeat adolescent birth. Compared to women with no birth <18 years, those with first birth <18 years were less likely to have completed primary education (16.3% versus 32.2%, p<0.001), more likely to be illiterate (55.0% versus 44.0%, p<0.001), to report challenges seeking healthcare (67.6% versus 61.8%, p=0.002), and had higher mean number of births by age 40 years (6.6 versus 5.3, p<0.001). Among women married at time of survey, those with birth <18 years had older husbands (p<0.001) who also had lower educational attainment (p<0.001). Educational attainment, household wealth score, total number of births, and under-five mortality among women with one adolescent birth were similar, and sometimes better, than among those with no birth <18 years.

Conclusions

For peer review only

For peer review only - http://bmjopen.bmj.com/site/about/guidelines.xhtml

Results suggest life-long adverse socioeconomic and reproductive outcomes among women with adolescent birth, primarily in the category with repeat adolescent birth. While our results might be birth-cohort specific, they underscore the need to support adolescent mothers to have the same possibilities to develop their potentials, by supporting school continuation and prevention of further unwanted pregnancies.

Strengths and limitations of this study

- We used a nationally-representative sample of women in Uganda and assessed the extent of differential recall in reporting of adolescent births (which was minimal).
- The cross-sectional nature of the data and our descriptive analysis did not allow for assessment of causality.
- The results may largely apply to this cohort due to societal changes and advancements in protection of women but, this is a critical starting point to examine long term impact of early birth and repeat adolescent birth.

Funding: This work was supported through the Developing Excellence in Leadership, Training and Science (DELTAS) Africa Initiative grant # DEL-15-011 to THRiVE-2. The DELTAS Africa Initiative is an independent funding scheme of the African Academy of Sciences (AAS)'s Alliance for Accelerating Excellence in Science in Africa (AESA) and supported by the New Partnership for Africa's Development Planning and Coordinating Agency (NEPAD Agency) with funding from the Wellcome Trust grant # 107742/Z/15/Z and the UK government. The views expressed in this publication are those of the author(s) and not necessarily those of AAS, NEPAD Agency, Wellcome Trust or the UK government.

For peer review only - http://bmjopen.bmj.com/site/about/guidelines.xhtml

Competing interests: None declared.

Introduction

The sub-Saharan Africa region continues to have the worlds' highest burden of adolescent births, accounting for 95% of the 12 million births occurring to 15-19-year-old girls each year globally (1-5). Just as in other parts of sub-Saharan Africa, the levels of adolescent childbearing in Uganda remain unacceptably high: 24.8% of women aged 15-19 years have begun childbearing (pregnant or have given birth), levels similar to 15 years ago (6-9). According to the 2016 Uganda Demographic and Health Survey (DHS), 28.4% of women aged 20-24 years had an adolescent birth before age 18, a decline from 41.7% in 1988/89. Among women aged 20-24 years for whom the first birth occurred before age 18, the percentage reporting repeat adolescent birth (first birth <18, and another birth before the age of 20 years) did not decline in 30 years (58.9% in 1988/89, 55.6% in 2016) (10).

Adolescence (ages 10-19) is characterized by rapid physical, social, cognitive and emotional development that lays the foundation for an individual's health and wellbeing across the life course. As noted by Patton et al. (2018), adolescence is a period when individuals acquire health, social, cultural, financial and educational assets, whose effects can spill over to the next generation (11).

For peer review only

Global evidence shows that childbirth in adolescence, especially before the age of 18 years, is associated with poor social, economic and health outcomes for both the girl and her offspring (12-16). The health challenges associated with adolescent birth include obstructed labor, postpartum hemorrhage, preterm birth, fistulae, sepsis, and infant death among others (5, 12, 17). In addition, adolescent childbirth can negatively affect socio-economic outcomes leading to school dropout, limited cash income, and forced/early marriage (18-21). Married adolescents are more likely than those unmarried to initiate childbearing early, permanently drop out of school, and lack autonomy over their sexuality and reproduction due to patriarchal control (22-24). Younger adolescent girls are more prone to the negative outcomes due to their immature physiological state, poor economic independence, and developing cognition (25, 26). Having repeat pregnancies during adolescence can further exacerbate the risk of such negative outcomes (27, 28).

While the immediate effects are relatively described, there is limited evidence of the long-term socio-economic and reproductive health outcomes following early initiation of childbearing, including the effects of repeat adolescent birth, in Uganda and other low-income countries (29, 30). The available information, mainly from high-income countries, suggests life-long persistence of socio-economic disadvantage such as low educational attainment, poor earnings, and less stable marriages following an adolescent birth (31-34). A Population Council and Women Deliver report explored the short- and long-term socio-economic (employment and cash earnings) impact of having a child <18 years using the most recent DHS data sets from 43 LMICs including the 2016 Uganda DHS (34). This report suggested that economic disadvantages of adolescent childbirth persist over the entire lifetime for women. A study in Mexico found that women ages 25-64 years who had a birth between ages 15-19 years had lower educational attainment and lower income

For peer review only

For peer review only - http://bmjopen.bmj.com/site/about/guidelines.xhtml

compared to those who did not (33). Global literature points to persistence of the negative effects of adolescent birth on a woman's social welfare and household, including into the next generations (2, 5, 12, 35). A woman's agency and decision-making power can be negatively affected. For example, the resultant low educational and wealth attainment following early birth (5) is associated with high unmet need for contraception (36). In the long term, women who start childbirth during adolescence tend to end up with older partners who have lower educational and socio-economic attainment (32, 37, 38). However, these studies did not disaggregate outcomes among women who had *repeat* adolescent birth following the first birth <18 years, nor did they assess outcomes related to women's life-long reproductive health.

In response to this gap, this paper seeks to investigate the socio-economic and reproductive outcomes among women in Uganda toward the end of reproductive life-course (40-49 years) according to their adolescent birth history (no birth <18 years, first birth <18 years, repeat births <20 years). We hypothesize that having an adolescent birth before the age of 18 years leads to lifelong disruption in the acquisition of socio-economic resources and wellbeing. Further, we hypothesize that repeat adolescent births (first birth before the age of 18 and second before the of age 20 years) further worsens these outcomes. Given the link between early marriage and childbirth and its potential influence on later life outcomes (32, 39), we also stratified outcomes by marital status among women with first birth <18 years (comparing those with and without repeat adolescent birth).

For peer review only - http://bmjopen.bmj.com/site/about/guidelines.xhtml

Methods

Data sources and population

We used data from the 2016 Uganda DHS individual women's dataset. The DHS are nationally representative cross-sectional surveys where multi-stage cluster sampling is conducted. All the geographic regions of the country were represented. The DHS are conducted every five years in Uganda and collect information on population health along with socioeconomic and demographic characteristics. All women aged 15-49 years in sampled households for the individual women's dataset provided self-reported information about their live births. The interviewer-administered questionnaires used were translated into local languages and pre-tested prior to data collection.

The study population included women aged 40-49 years at the time of the 2016 survey, whom we categorized into three adolescent birth histories: 1) no birth <18 years; 2) first birth <18 years and no additional births <20 years; and 3) first birth <18 years and one or more additional births <20 years (repeat adolescent birth). We included live births rather than pregnancies and analyzed all women, including those who never gave birth, given that some pregnancies may end in miscarriage/abortion and others in stillbirths and therefore, the later life outcomes may not be impacted by this. To validate women's, recall of adolescent childbearing, we conducted a sensitivity analysis comparing the childbirth patterns of women born in 1967-1976 as reported in the 1995 Uganda DHS to the same birth cohort of women as reported in 2016 (Supplementary table 1). We found similar estimates of first birth <18 years and repeat and no repeat adolescent births, indicating similar estimates of adolescent birth history among two representative samples of women from the same birth cohort in 1995 versus 2016.

Measures

For peer review only - http://bmjopen.bmj.com/site/about/guidelines.xhtml

157 *Outcome variables:* We assessed two main categories of later life outcomes: socio-economic and reproductive health-related. Supplementary table 2 contains the different variables assessed and their definitions. Socio-economic outcomes included: women's educational attainment (education level, means years of education); literacy; household wealth score (calculated using the household assets and presented as a linear index) (40); and receiving cash income at time of the 2016 Uganda DHS. Reproductive health outcomes included: number of live births by exact age 40 years; under-five mortality rate among any of the women's children; unmet need for contraception at the time of survey; and report of challenge seeking health care.

We further analyzed outcomes measured only in the sub-sample of women who were married or in a union at the time of the 2016 survey, including: age difference with husband; husband's education level (categorized in the same way as for women); and several measures of empowerment based on women's reports of whether they 1) solely decide on how their own earnings are spent, 2) are part of a marriage with other wives (polygamy), and 3) decide about their own health care.

Background variables included*:* women's area of residence (urban or rural); geographic region (Central, Eastern, Northern and Western); religion (Anglican, Catholic, Muslim, Other); marital status at survey (currently married/in union, divorced/separated, never in union); and the mean age (years) at survey, first sex, first birth, and at first union (if ever married/in union).

Statistical analysis

We began with exploratory data analysis to assess the distribution in outcomes and background characteristics and examine missingness. Next, we calculated the percentage of women according to their adolescent birth history (with vs. without first birth <18 years, and with vs. without repeat

For peer review only - http://bmjopen.bmj.com/site/about/guidelines.xhtml

adolescent birth). Column percentages of the later life outcomes and their 95% confidence intervals for each category of adolescent birth history were presented. We used the two-sample test of proportions to test for differences in the two column proportions for each later life outcome, and linear regression to compare group means for continuous variables. We further used Poisson regression to compare under-five mortality rates, estimating incidence risk ratios (IRR) and associated p-values. We described later life outcomes by adolescent birth history among all women and among the subsample of women who were married/in a union at time of survey (for whom partner information was available). We further described later life outcomes in a sub-analysis among women with and without repeat adolescent birth by marital status at first birth. For this sub-analysis, we included variables with p-value <0.05 between women with and without repeat adolescent births. Information on marital status at first birth were missing for N=95 (N=10 with no repeat adolescent birth and N=85 with repeat adolescent birth); these observations were therefore excluded from the sub-analysis.

We used survey weights, stratification and clustering to adjust for the complex survey design and non-response. All analyses were conducted using STATA version 12.0, StataCorp LP, Texas.

Patient and public involvement

For this study, we utilized secondary data and there was therefore no direct patient or public involvement. However, the study objectives and design were developed in collaboration with the Reproductive Health Division of the Ugandan Ministry of Health for whom adolescent fertility is a key research priority. The study builds on a previous landscape analysis of adolescent health needs conducted by the Ministry of Health, which indicated information gaps related to the long-

For peer review only

For peer review only - http://bmjopen.bmj.com/site/about/guidelines.xhtml

term effects of adolescent childbearing, especially for repeat adolescent birth. The results from the study will be disseminated to different stakeholders at the regional and national level.

Ethics

We obtained permission to use the datasets from the DHS program. During the survey data collection process, implemented by the Uganda Bureau of Statistics. written informed consent was obtained from all the respondents.

Results

From the analytical sample of 2,814 women aged 40-49 years in the 2016 Uganda DHS, 36.2% had their first birth <18 years (Figure 1). Among these, 85.9% reported a repeat adolescent birth (31.1% of all women). Similar proportions were observed for women who were married at the time of survey: 36.4% reported a first birth <18 years and of these, 87.1% reported a repeat adolescent birth (31.7% of all married women).

Table 1 shows the background characteristics of women in the 2016 sample based on their adolescent birth history. Most resided in rural areas (79.1%), belonged to the two main Christian religious faiths (Catholic and Anglican, 86.6%), and had ever been married or in a union (98%). The background characteristics were similar for women with and without first birth <18 years in terms of region, residence, religion, marital status, and mean age at survey. However, the mean age at first sex, first birth and first union differed by adolescent birth history. Women with first birth < age 18 reported earlier age at sexual debut (14.4 years vs. 17.3 years), and had their first birth and union earlier (15.5 and 16.3 years) compared to those who began childbearing after age

For peer review only - http://bmjopen.bmj.com/site/about/guidelines.xhtml

18. In contrast. women with repeat adolescent birth entered into marriage/union around the same age (15.9 years) as their first birth (15.5 years), whereas for those without repeat adolescent birth, first marriage or union (18.3 years) happened on average two years after their first birth (16.1 years). The average age at first birth for all women was 19.0 years whereas it was 15.5 years among those with first birth <18 years.

Later life outcomes by adolescent birth history

Looking at women all women aged 40-49 years (N=2814), results from bivariate analysis indicated that those who had their first birth <18 had significantly lower mean years of schooling (3.6 vs. 5.0 years, p<0.001), were less likely to only have completed primary school or higher (16.3% versus 32.2%, p<0.001), and more likely to be illiterate (55.0% vs 44.0%, p<0.001) compared to those without a birth <18 years (Table 2). Further, they also had lower household wealth score (p=0.043), were more likely to report health care seeking challenges (p=0.002), and had a higher mean number of live births by exact age 40 years (6.6 versus 5.3, p<0.001). There were no differences between the two groups in children's under-five mortality rate.

Among the 1,020 women with first birth <18 years, those with repeat adolescent birth were less likely than those without such experiences to have completed at least primary education (14.2% vs. 28.9%, p<0.001), to have lower mean years of education (3.4 vs. 4.7 years, p<0.001), household wealth scores (-0.003 vs. 0.020, p=0.032), more likely to be illiterate (57.1% vs. 41.9%, p<0.001), and to have higher mean number of live births by age 40 (7.0 versus 4.0, p<0.001). Further, the under-five mortality rate for children born to women with repeat adolescent births was higher than for those born to women with no repeat adolescent birth (IRR=1.36, 95% CI 1.06-

For peer review only

For peer review only - http://bmjopen.bmj.com/site/about/guidelines.xhtml

1.75). Health care seeking challenges and unmet need for contraceptives were, however, similar when comparing women with and without repeat adolescent births.

Analysis of a subsample of women 40-49 years married at time of 2016 survey

We further conducted analysis among a subsample of women who were married at time of the 2016 survey (N=2,022). Compared to women with no birth <18 years, those with first birth <18 years had partners/husbands who were older (7.6 versus 5.4 years older, p<0.001) and with lower education level (54.1% versus 61.2% primary school or above, p=0.002) (Table 3). Further, more women with first birth <18 years had control over how their own earnings are spent (p<0.001). There were no differences in outcomes regarding being in polygamous marriage and non-inclusion in decision-making about women's own healthcare. Among the 737 married women who had a first birth <18 years, there were no other differences in outcomes observed by whether they had repeat adolescent births.

Analysis of women with and without repeat adolescent births, by marital status at first birth

We also conducted analysis of later life outcomes among women with and without repeat adolescent birth by their marital status at first birth, including variables that were significantly associated with repeat adolescent birth (p<0.05) among all women aged 40-49 years (Table 2). The proportion married at first birth was higher for those with vs. without repeat adolescent birth (76.4% vs. 63.4%, p=0.001) (Table 4). Results showed no differences in later life outcomes based on marital status among women without repeat adolescent births. However, among women with repeat adolescent birth, those who were married at first birth had lower mean years of schooling

For peer review only

For peer review only - http://bmjopen.bmj.com/site/about/guidelines.xhtml

(3.2 vs. 3.9 years, p=0.017) and were more likely to be illiterate (59.7% vs. 49.3%, p=0.012) compared to those who were not married at the time.

Discussion

We investigated the socio-economic and reproductive health outcomes among Ugandan women towards the end of their reproductive life using data from the most recent Uganda DHS collected in 2016. We compared women's outcomes according to their adolescent birth histories: those with and without first birth <18 years, and among those with first birth <18 years, those with and without repeat adolescent births (two or more births before age 20). For the latter groups, we also examined differences in later life outcomes by marital status at the point of the first adolescent birth.

Our findings highlight three key points. First, women who had their first birth <18 years reported more negative socio-economic and reproductive health outcomes in later life than those without such experiences. These differences appear to be driven by the largest subgroup of women with repeat adolescent birth, who had poorer educational attainment, lower household wealth and empowerment, as well as higher fertility and under-five infant mortality compared to the other groups. This finding aligns with previous research highlighting adolescent childbirth as both a risk-factor for, and consequence of, poverty and low education (5, 41, 42). School discontinuation due to pregnancy is a persistent problem (42), and once a girl has given birth, she needs social, psychosocial and economic support to go back to school and complete her secondary education (5). Our findings further suggest that the risk of school discontinuation was greatest for girls who were married at first birth and went on to have repeat adolescent births, compared to their unmarried counterparts. It is possible that married adolescents with repeat adolescent birth are

For peer review only

For peer review only - http://bmjopen.bmj.com/site/about/guidelines.xhtml

taken out of school entirely (34, 42), underscoring the need to prevent early marriage in a country where marriage before age 18 remains common (40%) (43, 44), and thus a risk factor for repeat adolescent birth and its negative later life outcomes.

Secondly, women with an adolescent birth, and especially with repeat adolescent birth, were more likely to be socio-economically disadvantaged in later life – confirming the limited evidence on poor economic trajectories following early childbearing (33). For example, the Population Council and Women Deliver analysis of data from 43 low- and middle-income countries found that adolescents who gave birth before age 18 were significantly less likely to be earning cash, although there were no differences in employment (45). This suggests that low educational attainment and the burden of caring for many children ultimately lowered women's agency and chances of economic empowerment. This position is further supported by our finding of similar, or even better, socioeconomic outcomes among women with one adolescent birth compared to those with no birth <18 years; the lower mean number of children provided avenues for these women to pursue economic activities. Having a partner with a low educational attainment, as was the case for those with repeat adolescent births, may further have worsened the negative economic cycle. Indeed, women's opportunity for economic empowerment are not only linked to their educational attainment and literacy, but to the status of the spouse, which in turn impact household wealth scores (34, 46, 47). Our findings thus confirm the well-demonstrated link between poverty, low education and high fertility globally (5), calling for renewed efforts to alleviate household poverty and maintain adolescent girls in school.

We also found that women with first birth <18 years were more likely to report greater decision-making power over their own earnings, underscoring the results from the Population Council and Women Deliver report which found that adolescent birth was associated with more

For peer review only - http://bmjopen.bmj.com/site/about/guidelines.xhtml

control over own economic assets. This suggests that women with an adolescent birth might learn to be self-reliant and independent, providing a potential avenue for interventions to economically empower these women.

Third, our study results suggest that having a large completed family size is driven not only by an early initiation of childbearing, but by repeat adolescent births. Although the adolescent fertility rate has declined in Uganda, it remains high at 132 births per 1,000 women aged 15-19 years (9). This is reflected in how cultural norms in Uganda promote early marriage and childbearing, despite the legislation against early marriage (39, 42, 48, 49). The household poverty makes it worse, as girls are forced to discontinue schooling and get sent off into union in exchange for bride price (44, 50, 51). Low access and high unmet need for contraceptives among adolescent girls and young women in Uganda further restrict girls' reproductive agency in a heavily patriarchal context (52). Even in situations where an adolescent girl may want to use contraception, the partner or health worker may object to allow her to access the method (53). Further, abortion in Uganda is restricted to particular medical conditions, forcing girls facing an unintended pregnancy to either give birth or risk unsafe abortion (54, 55).

For peer review only

The care of a child born to an adolescent girl takes broadly two patterns in Uganda and this cultural position needs to be factored in when viewing outcomes of women with early birth; those in and out of union. When a woman gives birth within marriage, the responsibility of raising the child is usually taken on by the man or his family with the support of the woman (56). In contrast, when they conceive outside union, the woman and child is usually sent off by the family to live with the man responsible for the pregnancy (18, 57, 58). In this situation, the man is assumed to take on the responsibility of raising the child. These cultural norms have remained essentially unchanged over

For peer review only - http://bmjopen.bmj.com/site/about/guidelines.xhtml

the last four decades (50) despite advocacy for more paternal involvement in the care of children, most especially in scenarios of no union/marriage. Women out of union would most probably have been left with the entire burden of the offspring(s).

Suggestions for policy change and future research

Public health interventions are needed to prevent repeat adolescent birth and potential negative life-course outcomes via improved access to continued schooling and higher education, and effective contraceptive services. Development of a Uganda school continuation policy for adolescent mothers needs to be fast tracked followed by: wide dissemination, sensitization of the communities and parents, and enforcement at school level (49-52, 59). Contraception information and services need to be made more accessible to all adolescents in a manner that promotes their autonomy. Further, adolescent women should be protected from early marriage by keeping them in school and strengthening the legislation against early marriage (48, 50, 60). Our findings call for more in-depth research to explore and define what women without repeat adolescent birth did that enabled them to recuperate from the effects of early initiation of birth. Further, qualitative studies are needed to explore motivators and circumstances for repeat adolescent birth in sub-Saharan Africa. Finally, we suggest that secondary level completion among adolescent mothers be investigated further among younger cohorts, especially now that the Ministry of Education in Uganda, and many other countries in sub-Sahara Africa, permit girls to continue/resume schooling following pregnancy and childbirth (41).

Strength and limitations

For peer review only

For peer review only - http://bmjopen.bmj.com/site/about/guidelines.xhtml

This study utilized data from a large nationally representative sample containing a variety of variables. However, the cross-sectional data cannot support causal associations between adolescent childbirth and the outcomes examined. While our assessment of differential reporting of adolescent childbirth did not show variation on a population level, the results may be influenced by reporting and recall bias due to the self-reported data. Women may also have given socially desirable responses regarding the age at first birth (61). In addition, some risk factors may have been present right from early childhood. Due to this reverse causality, we cannot be certain of what came first; the negative outcomes or the birth. Nonetheless, this nationally representative survey data is a starting point to investigate later life outcomes in the absence of a prospective cohort in LMIC setting. Additionally, circumstances and opportunities available to women approximately 20 years ago may have altered and therefore, it may not fully depict the current situation in Uganda.

Conclusion

Findings from this study, while not necessarily causal, suggest that among women in Uganda, giving birth before age 18 years is linked to lower educational attainment, household wealth, and higher fertility and under-five infant mortality outcomes later in life. Further, they tend to have spouses who are older and of low socio-economic attainment. This pattern is especially strong for women who have repeat adolescent births, and particularly for those who were married at first birth. Women who begin childbearing before age 18, but do not proceed to have a repeat adolescent birth – despite being a minority (5%) – appear to have recovered from any negative effects, and in some instances had better outcomes than those with no birth <18 years. This suggests persistence of the negative outcomes thereby underscoring the need to not only prevent early adolescent birth but, prevent repeat adolescent births.

For peer review only - http://bmjopen.bmj.com/site/about/guidelines.xhtml

Figure 1: Adolescent birth history among Uganda women aged 40-49 years at the 2016 Uganda DHS

References:

1. Temmerman M. Adolescent mothers: too young to be neglected. The Lancet Child & Adolescent Health. 2017;1(3):164-6.
2. Timæus IM, Moultrie TA. Teenage Childbearing and Educational Attainment in South Africa. Studies in Family Planning. 2015;46(2):143-60.
3. Neal S, Matthews Z, Frost M, Fogstad H, Camacho AV, Laski L. Childbearing in adolescents aged 12-15 years in low resource countries: a neglected issue. New estimates from demographic and household surveys in 42 countries. Acta Obstet Gynecol Scand. 2012;91(9):1114-8.
4. Yakubu I, Salisu WJ. Determinants of adolescent pregnancy in sub-Saharan Africa: a systematic review. Reproductive health. 2018;15(1):15.
5. WHO. Adolescent pregnancy. Fact sheet. World Health Organization; 2020 31 January 2020.
6. Neal S, Ruktanonchai C, Chandra-Mouli V, Matthews Z, Tatem AJ. Mapping adolescent first births within three east African countries using data from Demographic and Health Surveys: exploring geospatial methods to inform policy. Reprod Health. 2016;13(1):98.
7. Neal SE, Chandra-Mouli V, Chou D. Adolescent first births in East Africa: disaggregating characteristics, trends and determinants. Reproductive health. 2015;12(1):13.
8. Wado YD, Sully EA, Mumah JN. Pregnancy and early motherhood among adolescents in five East African countries: a multi-level analysis of risk and protective factors. BMC pregnancy and childbirth. 2019;19(1):59.
9. UBOS. Uganda Demographic and Health Survey 2016. Kampala, Uganda and Rockville, Maryland, USA: UBOS and ICF; 2018 2018.
10. Amongin D, Nakimuli A, Hanson C. Time trends in and factors associated with repeat adolescent birth in Uganda: Analysis of six demographic and health surveys. 2020;15(4):e0231557.
11. Patton GC, Olsson CA, Skirbekk V, Saffery R, Wlodek ME, Azzopardi PS, et al. Adolescence and the next generation. Nature. 2018;554(7693):458-66.
12. Loredo-Abdala A, Vargas-Campuzano E, Casas-Munoz A, Gonzalez-Corona J, Gutierrez-Leyva CJ. [Adolescent pregnancy: its causes and repercussions in the dyad]. Rev Med Inst Mex Seguro Soc. 2017;55(2):223-9.
13. Pérez MM, Barquero ML, Sánchez ML, De L, Los Rios Gestoso GTR, Aso CM, et al. CONSEQUENCES OF TEEN PREGNANCY.
14. Pinto ESJL, Surita FG. Pregnancy in Adolescence - A Challenge Beyond Public Health Policies. Rev Bras Ginecol Obstet. 2017;39(2):41-3.
15. Blanc AK, Winfrey W, Ross J. New findings for maternal mortality age patterns: aggregated results for 38 countries. PloS one. 2013;8(4):e59864.

For peer review only

For peer review only - http://bmjopen.bmj.com/site/about/guidelines.xhtml

16. Nove A, Matthews Z, Neal S, Camacho AV. Maternal mortality in adolescents compared with women of other ages: evidence from 144 countries. The Lancet Global Health. 2014;2(3):e155-e64.

17. United Nations DoEaSA, Population Division. World Fertility 2019: Early and later childbearing among adolescent women. 2020.

18. Atuyambe L, Mirembe F, Johansson A, Kirumira EK, Faxelid E. Experiences of pregnant adolescents--voices from Wakiso district, Uganda. Afr Health Sci. 2005;5(4):304-9.

19. Mollborn S. Teenage Mothers Today: What We Know and How It Matters. Child Development Perspectives. 2017;11(1):63-9.

20. Diaz CJ, Fiel JE. The effect (s) of teen pregnancy: Reconciling theory, methods, and findings. Demography. 2016;53(1):85-116.

21. Ranchhod V, Lam D, Leibbrandt M, Marteleto L. Estimating the effect of adolescent fertility on educational attainment in Cape Town using a propensity score weighted regression. 2011.

22. Adesina M, Daha M, Ajobiewe A, Oladele RI, Olufadewa I, Ayorinde T. The High Proportion of Child Brides in Africa: Implications for The Girl Child and The Society. 2020.

23. Sunmola AK, Olaosebikan JS, Adeusi TJ. High fertility level in Sub-Saharan Africa: implication for reaping and optimizing demographic dividend. International Journal of Community Medicine and Public Health. 2020;7(9):3698.

24. Delprato M, Akyeampong K, Dunne M. Intergenerational education effects of early marriage in Sub-Saharan Africa. World Development. 2017;91:173-92.

25. Leftwich HK, Alves MVO. Adolescent Pregnancy. Pediatric Clinics of North America. 2017;64(2):381-8.

26. Chen X-K, Wen SW, Fleming N, Demissie K, Rhoads GG, Walker M. Teenage pregnancy and adverse birth outcomes: a large population based retrospective cohort study. International journal of epidemiology. 2007;36(2):368-73.

27. Maravilla JC, Betts KS, Alati R. Increased risk of maternal complications from repeat pregnancy among adolescent women. International Journal of Gynecology & Obstetrics. 2019.

28. Maravilla JC, Betts KS, Alati R. Trends in repeated pregnancy among adolescents in the Philippines from 1993 to 2013. Reproductive health. 2018;15(1):184.

29. Fall CH, Sachdev HS, Osmond C, Restrepo-Mendez MC, Victora C, Martorell R, et al. Association between maternal age at childbirth and child and adult outcomes in the offspring: a prospective study in five low-income and middle-income countries (COHORTS collaboration). The Lancet Global Health. 2015;3(7):e366-e77.

30. Ellis-Sloan K. Teenage mothers in later life: Time for a second look. Journal of Adolescence. 2019;77:98-107.

31. Lounds Taylor J. Midlife impacts of adolescent parenthood. Journal of family issues. 2009;30(4):484-510.

32. Hoffman SD, Maynard RA. Kids having kids: Economic costs & social consequences of teen pregnancy: The Urban Insitute; 2008.

33. Arceo-Gómez EO, Campos-Vázquez RM. Teenage pregnancy in Mexico: evolution and consequences. Latin american journal of economics. 2014;51(1):109-46.

34. Women Deliver and The Population Council. Having a child before becoming an adult. Exploring the economic impact in a multi-country Analysis. Report. New York: Women Deliver; 2019 2019.

35. Aitken Z, Hewitt B, Keogh L, LaMontagne AD, Bentley R, Kavanagh AM. Young maternal age at first birth and mental health later in life: Does the association vary by birth cohort? Social Science & Medicine. 2016;157:9-17.

36. Ethiopia K. What it takes: Meeting unmet need for family planning in East Africa. Policy. 2018.

For peer review only

For peer review only - http://bmjopen.bmj.com/site/about/guidelines.xhtml

37. Card JJ, Wise LL. Teenage mothers and teenage fathers: The impact of early childbearing on the parents' personal and professional lives. Family planning perspectives. 1978;10(4):199-205.
38. Ermisch J. Does a'teen-birth'have longer-term impacts on the mother? suggestive evidence from the British Household Panel Study. ISER Working Paper Series; 2003.
39. Walker J-A. Early marriage in Africa–trends, harmful effects and interventions. African journal of reproductive health. 2012;16(2):231-40.
40. Filmer D, Pritchett LH. Estimating wealth effects without expenditure data—or tears: an application to educational enrollments in states of India. Demography. 2001;38(1):115-32.
41. Watch HR. LEAVE NO GIRL BEHIND IN AFRICA: Discrimination in Education against Pregnant Girls and Adolescent Mothers. 2018.
42. Wodon Q, Nguyen MC, Tsimpo C. Child Marriage, Education, and Agency in Uganda. Feminist Economics. 2016;22(1):54-79.
43. Male C, Wodon Q. Basic profile of child marriage in Uganda. 2016.
44. Petroni S, Steinhaus M, Fenn NS, Stoebenau K, Gregowski A. New findings on child marriage in sub-Saharan Africa. Annals of global health. 2017;83(5-6):781-90.
45. Wilkie GL, Leung K, Kumaraswami T, Barlow E, Simas TAM. Effects of Obstetric Complications on Adolescent Postpartum Contraception and Rapid Repeat Pregnancy. Journal of pediatric and adolescent gynecology. 2016;29(6):612-6.
46. Rasmussen B, Maharaj N, Sheehan P, Friedman HS. Evaluating the employment benefits of education and targeted interventions to reduce child marriage. Journal of Adolescent Health. 2019;65(1):S16-S24.
47. Kanjilal-Bhaduri S, Pastore F. Returns to education and female participation nexus: Evidence from India. The Indian Journal of Labour Economics. 2018;61(3):515-36.
48. Addaney M, Azubike O-O. Education as a Contrivance to Ending Child Marriage in Africa: Perspective from Nigeria and Uganda. Amsterdam LF. 2017;9:110.
49. Leerlooijer JN, Bos AE, Ruiter RA, Van Reeuwijk MA, Rijsdijk LE, Nshakira N, et al. Qualitative evaluation of the Teenage Mothers Project in Uganda: a community-based empowerment intervention for unmarried teenage mothers. BMC public health. 2013;13(1):816.
50. Bantebya GK, Muhanguzi FK, Watson C. Adolescent girls in the balance: Changes and continuity in social norms and practices around marriage and education in Uganda. Overseas Development Institute: London, UK. 2014.
51. Okwany A, Kamusiime A. Foregrounding the tensions and silences in education policies for student-mothers in Uganda and Kenya. Childhood in Africa. 2017;4(1).
52. Wodon QT, Male C, Onagoruwa AO, Savadogo A, Yedan A. The cost of not investing in girls: child marriage, early childbearing, low educational attainment for girls, and their impacts in Uganda. The World Bank; 2017.
53. Nalwadda G, Mirembe F, Byamugisha J, Faxelid E. Persistent high fertility in Uganda: young people recount obstacles and enabling factors to use of contraceptives. BMC public health. 2010;10(1):530.
54. Sully EA, Atuyambe L, Bukenya J, Whitehead HS, Blades N, Bankole A. Estimating abortion incidence among adolescents and differences in postabortion care by age: a cross-sectional study of postabortion care patients in Uganda. Contraception. 2018;98(6):510-6.
55. Atuyambe LM, Kibira SP, Bukenya J, Muhumuza C, Apolot RR, Mulogo E. Understanding sexual and reproductive health needs of adolescents: evidence from a formative evaluation in Wakiso district, Uganda. Reproductive health. 2015;12(1):1-10.
56. Ickes SB, Heymsfield GA, Wright TW, Baguma C. “Generally the young mom suffers much:” Socio-cultural influences of maternal capabilities and nutrition care in Uganda. Maternal & child nutrition. 2017;13(3):e12365.
57. Ninsiima AB, Leye E, Michielsen K, Kemigisha E, Nyakato VN, Coene G. “Girls Have More Challenges; They Need to Be Locked Up”: A Qualitative Study of Gender Norms and the

For peer review only

For peer review only - http://bmjopen.bmj.com/site/about/guidelines.xhtml

Sexuality of Young Adolescents in Uganda. International journal of environmental research and public health. 2018;15(2):193.
58. Kaye DK. Negotiating the transition from adolescence to motherhood: Coping with prenatal and parenting stress in teenage mothers in Mulago hospital, Uganda. BMC Public Health. 2008;8(1):1-6.
59. Birungi H, Undie C-C, MacKenzie I, Katahoire A, Obare F, Machawira P. Education sector response to early and unintended pregnancy: A review of country experiences in Sub-Saharan Africa. 2015.
60. Kalamar AM, Lee-Rife S, Hindin MJ. Interventions to prevent child marriage among young people in low-and middle-income countries: a systematic review of the published and gray literature. Journal of Adolescent Health. 2016;59(3):S16-S21.
61. Neal SE, Hosegood V. How Reliable Are Reports of Early Adolescent Reproductive and Sexual Health Events in Demographic and Health Surveys? Int Perspect Sex Reprod Health. 2015;41(4):210-7.

Table 1: Background characteristics of Uganda women 40-49 years by adolescent birth history; 2016 Uganda DHS

	**All women 40-49 years** *col% (95% CI)*	**No birth <18 years** *col% (95% CI)*	**1st Birth <18 years** *col% (95% CI)*	**Birth <18 years, no repeat** *col% (95% CI)*	**Birth <18 years, Repeat** *col% (95% CI)*
**Variables**	**N=2,814**	**N=1,795**	**N=1,020**	**N=144**	**N=876**
**Residence**					
Urban	20.9 (18.7-23.3)	21.0 (18.3-23.9)	20.8 (17.7-24.3)	23.7 (16.4-33.1)	20.3 (17.4-23.7)
Rural	79.1 (76.7-81.3)	79.0 (76.1-81.7)	79.2 (75.7-82.3)	76.3 (66.9-83.6)	79.7 (76.3-82.6)
**Region**					
Central	26.5 (22.3-31.1)	24.4) (20.2-29.3	30.1 (24.8-36.0)	31.4 (21.8-42.9)	29.9 (24.3-36.1)
Eastern	30.3 (26.1-34.9)	29.4 (25.1-34.1)	31.9 (26.8-37.6)	30.2 (21.4-40.6)	32.2 (26.9-38.1)
Northern	17.6 (14.6-21.1)	18.3 (15.1-22.1)	16.5 (13.2-20.2)	18.1 (12.3-26.0)	16.2 (12.9-20.0)
Western	25.6 (21.7-29.8)	27.9 (23.5-32.7)	21.5 (17.6-26.0)	20.3 (13.8-28.9)	21.7 (17.6-26.4)
**Religion**					
Anglican	44.6 (42.0-47.2)	44.8 (41.9-47.9)	44.1 (40.1-48.1)	39.4 (30.6-48.9)	44.8 (40.6-49.2)
Catholic	42.0 (39.2-44.9)	43.2 (39.9-46.5)	40.0 (36.2-43.9)	48.0 (38.9-57.2)	38.7 (34.6-43.0)
Muslim	10.5 (8.8-12.6)	9.1 (7.2-11.4)	13.0 (10.4-16.2)	9.2 (5.3-15.7)	13.7 (10.8-17.1)
Other	2.9 (2.2-3.8)	2.9 (2.1-4.0)	2.9 (1.8-4.5)	3.4 (1.2-9.2)	2.8 (1.7-4.5)
**Marital status at survey**					
Never in union	2.0 (1.5-2.7)	2.4 (1.7-3.4)	1.3 (0.7-2.3)	0.3 (0.0-2.0)	1.4 (0.8-2.6)
Currently in union/marriage	71.8 (69.9-73.7)	71.6 (69.1-73.9)	72.3 (69.0-75.4)	66.4 (57.1-74.5)	73.3 (69.8-76.5)
Formerly in union/marriage	26.2 (24.3-28.1)	26.0 (23.7-28.5)	26.4 (23.4-29.7)	33.4 (25.2-42.6)	25.3 (22.1-28.7)

For peer review only - http://bmjopen.bmj.com/site/about/guidelines.xhtml

**Mean age at survey (sd)**	43.9 (2.9)	44.1 (2.8)	43.8 (2.9)	43.5 (2.9)	43.9 (2.9)
**Mean age at first sex (sd)**	16.3 (2.8)	17.3 (2.8)	14.4 (1.5)	15.1 (1.4)	14.3 (1.5)
**Mean age at 1st birth (sd)**	19.0 (3.9)	21.1 (3.3)	15.5 (1.5)	16.1 (1.2)	15.5 (1.5)
**Mean age at 1st union, if ever married (SD)**	18.9 (5.3)	20.3 (4.9)	16.3 (5.0)	18.3 (5.8)	15.9 (4.8)

For peer review only

Table 2: Socioeconomic and health-related life course outcomes by adolescent birth history among Uganda women 40-49 years, 2016 Uganda DHS (N=2815)

	**All women 40-49 years**			**Women with first birth <18 years**		
	No birth <18 years **(N=1795)**	Birth <18 years **(N=1020)**		No repeat adolescent birth **(N=144)**	Repeat adolescent birth **(N=876)**	
**Variables**	% (95% CI)	% (95% CI)	***p***	% (95% CI)	% (95% CI)	*p*
**Education level**						
Complete primary and above	32.2 (29.3-35.3)	16.3 (13.7-19.2)	<0.001	28.9 (21.2-37.9)	14.2 (11.6-17.3)	<0.001
**Mean years in school (SD)**	5.0 (4.4)	3.6 (3.2)	<0.001	4.7 (3.4)	3.4 (3.1)	0.001
**Literacy**						
Illiterate (cannot read at all)	44.0 (40.9-47.1)	55.0 (50.8-59.1)	<0.001	41.9 (32.7-51.8)	57.1 (52.8-61.4)	<0.001
**Household wealth score**	0.009 (0.100)	0.001 (0.085)	0.043	0.020 (0.099)	-0.003 (0.082)	0.032
**Receiving cash income at survey (yes)**	70.0 (67.0-72.8)	70.5 (67.1-73.7)	0.780	71.3 (61.7-79.2)	70.4 (66.7-73.8)	0.826
**Challenge seeking health care (yes)**	61.8 (58.6-64.9)	67.6 (63.9-71.1)	0.002	62.0 (52.2-70.9)	68.5 (64.5-72.3)	0.123
**Mean No. of live births by exact age 40 years (SD)**	5.3 (2.1)	6.6 (1.7)	<0.001	4.0 (1.6)	7.0 (1.3)	<0.001
**U5 mortality rate**	124.6	140.1	P=0.341	104.8	142.9	P=0.016
**Unmet need for contraception**	19.8 [17.8,21.9]	20.2 (17.4-23.4)	0.799	20.1 (13.0-29.6)	20.2 [17.1,23.8]	0.978

* Under5mortality: Divided by 1000 live births. P-value obtained for the IRR that compared the mortality rate between 2 groups. P=0.016, IRR was 1.36 (95% CI 1.06-0.75).

Table 3: Later life outcomes related to partner characteristics among a subsample of married women 40-49 (2016 Uganda DHS, N=2022)

	All women 40-49 years			Women with first birth <18 years		
	**No birth <18 years (N=1285)**	**Birth <18 years (N=737)**		**No repeat adol. birth (N=096)**	**Repeat adol. birth (N=642)**	
**Variables**	% (95% CI)	% (95% CI)	*p*	% (95% CI)	% (95% CI)	*p*
**Mean age difference with partner/husband- years (SD)**	5.4 (6.4)	7.6 (7.2)	<0.001	6.8 (7.1)	7.7 (7.2)	0.358
**Husband's education level**	N=1285	N=737		N=96	N=642	
Primary	54.1 (50.5-57.7)	61.2 (56.9-65.3)	0.002	60.0 (48.6-70.5)	61.3 (56.8-65.6)	0.808
Secondary/higher	34.1 (30.7-37.7)	26.6 (22.9-30.6)	<0.001	31.7 (22.2-43.0)	25.8 (22.0-30.0)	0.222
**Women who solely decide on how her earnings are spent**	N=897<br>52.1 (48.3-55.9)	N=499<br>64.2 (58.8-69.3)	<0.001	N=68<br>64.8 (50.2-77.0)	N=499<br>64.1 (58.6-69.3)	0.910
**Women not included on decisions about:**						
Their own health care	18.8 (16.4-21.4)	20.9 (17.5-24.8)	0.252	15.5 (8.7-26.0)	21.8 (17.9-26.2)	0.157
**In marriage with other wives (polygamy)**	33.1 (29.8-36.6)	36.2 (32.3-40.2)	0.157	42.9 (31.1-55.6)	35.2 (31.1-39.4)	0.143

For peer review only - http://bmjopen.bmj.com/site/about/guidelines.xhtml

For peer review only

Table 4: Later life outcomes among women 40-49 years with and without repeat adolescent birth, by marital status at first birth (N=925)

	No repeat adolescent birth			Repeat adolescent birth		
	Not married at 1st birth (N=49)	Married at 1st birth (N=85)		Not married at 1st birth (N=186)	Married at 1st birth (N=604)	
**Variables**	% (95% CI)	% (95% CI)	***p***	% (95% CI)	% (95% CI)	***p***
**Education level**						
Complete primary and above	29.6 (17.5-45.4)	24.7 (15.7-36.5)	0.536	17.4 (12.1-24.4)	12.4 (9.7-15.8)	0.082
**Mean years in school (SD)**	4.7 (3.0)	4.2 (3.2)	0.458	3.9 (3.0)	3.2 (3.1)	0.017
**Literacy**						
Cannot read at all (illiterate)	40.3 (25.2-57.5)	44.4 (32.2-57.4)	0.644	49.3 (40.1-58.6)	59.7 (54.7-64.5)	0.012
**Household wealth scores** (SD)	0.005 (0.082)	0.024 (0.097)	0.320	0.004(0.085)	-0.008 (0.078)	0.097
**Mean No. of live births by exact age 40 years** (39.99) (SD)	4.0 (1.7)	4.2 (1.6)	0.602	7.0 (1.2)	7.0 (1.3)	0.963
**U5 mortality rate**/1000 live births	109.3	107.1	0.884	140.4	144.6	0.803

NB: * P-value of the IRR obtained from comparing the mortality rates. P=0.884 (IRR 0.98, 95%CI=0.75-1.28) and p=0.803 (IRR 1.03, 95% CI=0.82-1.30)

Abbreviations: BMI, Body Mass Index; DHS, Demographic and Health Surveys; FP, Family Planning; IRR, Incidence risk Ratio; LMIC, Low-and-Middle-Income Country; SD, Standard Deviation; STI, Sexually Transmitted Infection; U5, Under-five.

Author contributions:

DA conceived and designed the study, obtained permission to use data set from DHS program, conducted analysis, presented and interpreted results, drafted and finalized the article. AK conceived the study, conducted the literature review, reviewed and interpreted the results, and reviewed the article. ÖT conceived the study, drafted the analysis plan, reviewed and interpreted the results and reviewed the article. AN was involved reviewing the study design, the results and drafting the article. MN was involved in data analysis, presentation and interpretation of the results.

For peer review only

For peer review only - http://bmjopen.bmj.com/site/about/guidelines.xhtml

LA was involved in reviewing and interpreting the results and reviewing the manuscript. CH was involved in designing the study, the analysis, presenting and interpreting the results, drafting the article and reviewing the manuscript. LB was involved in conceiving the study, designing the analysis plan, data analysis, interpreting the results and substantially reviewing the article. All the authors reviewed and approved the manuscript. All the authors take responsibility for their contributions. None of the authors has competing interests.

Data sharing: Data used for this analysis can be accessed, with permission, from the Demographic and Health Survey program website https://www.dhsprogram.com/data/available-datasets.cfm

For peer review only

For peer review only - http://bmjopen.bmj.com/site/about/guidelines.xhtml

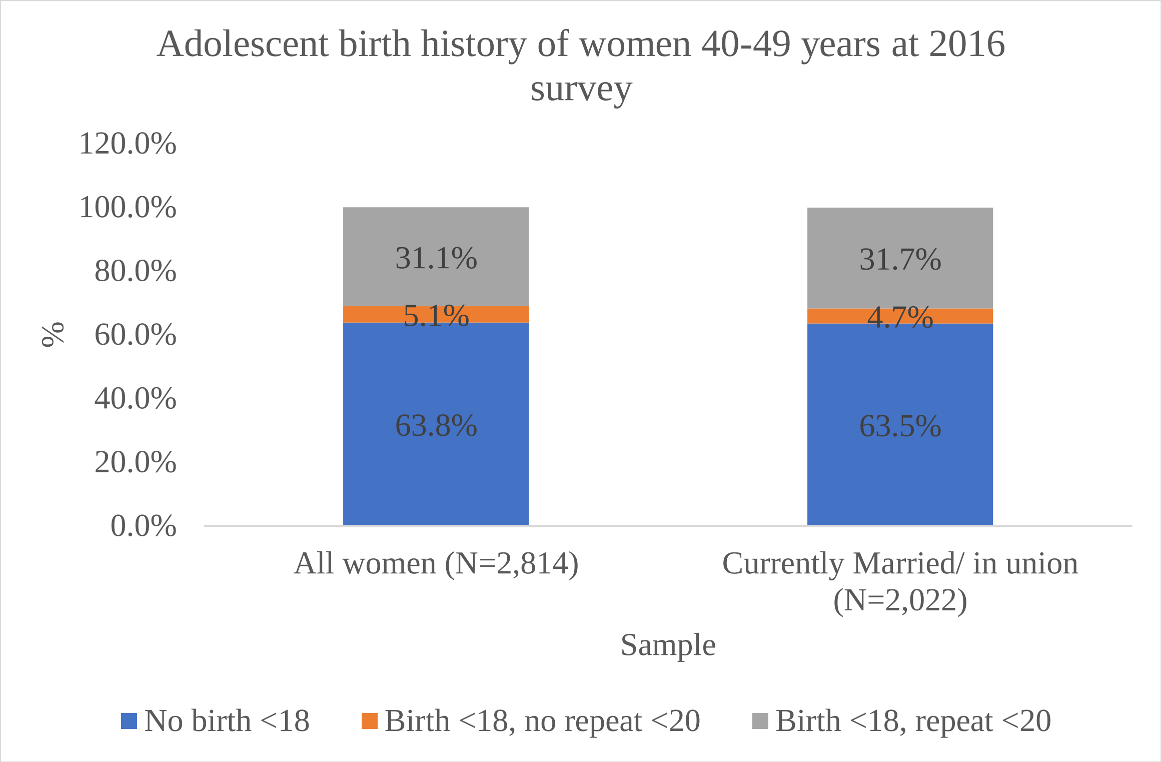

Figure 1: Adolescent birth history among Uganda women aged 40-49 years at the 2016 Uganda DHS

Supplementary table 1: Adolescent birth history among women born between 1967-1976 (1995 and 2016 UDHS)

Adolescent fertility pattern	UDHS 1995	UDHS 2016
	N=2,723	N=2,841
	% (95% CI)	% (95% CI)
**No birth <18**	61.0 (58.6-63.5)	63.4 (61.2-65.5)
**Birth <18, no repeat birth <20**	15.0 (13.6-16.5)	14.0 (12.6-15.5)
**Birth <18, repeat birth <20**	24.0 (22.0-26.1)	22.7 (20.9-24.6)

For peer review only

Supplementary table 2: The variable definitions/categorization for the analysis of the 2016 UDHS

Variable	Categories/definitions
**Residence**	• Rural<br>• Urban
**Region**	• Central (Kampala, Central 1 (South Buganda) & Central 2 (North Buganda))<br>• Eastern (Teso, Karamoja, Bugisu, Bukedi, & Busoga sub-regions)<br>• Northern (Lango, Acholi & West Nile sub-regions)<br>• Western (Bunyoro, Tooro, Ankole & Kigezi sub-regions)
**Religion**	• Anglican (Anglican & Pentecostal/born again/evangelical)<br>• Catholic<br>• Muslim<br>• Other (Seventh day Adventist, orthodox, Baptist, traditional, no religion, other, & Jehovah's Witness)
**Marital status at survey**	• Never in union<br>• Currently in union/marriage (married and living with partner)<br>• Formerly in union/marriage (widowed, divorced, and no longer living together/separated).
**Socioeconomic**	
**Educational attainment**	
Education level	• Incomplete primary (no education & incomplete primary)<br>• Complete primary and higher (Complete primary, incomplete secondary, complete secondary, and higher)
Mean number of years in school (education)	Maintained as a continuous variable
**Literacy**	• cannot read at all (illiterate)<br>• can read (able to read only parts of sentence & able to read whole sentence)<br>• no card with required language<br>• blind/visually impaired
**Receiving cash income at survey**	• Yes (cash only & cash and in-kind)<br>• No (not paid, in-kind only, & missing)

For peer review only

STROBE Statement—Checklist of items that should be included in reports of ***cross-sectional studies***

	Item No	Recommendation	Page No
**Title and abstract**	1	(*a*) Indicate the study's design with a commonly used term in the title or the abstract	1
		(*b*) Provide in the abstract an informative and balanced summary of what was done and what was found	3
**Introduction**			
Background/rationale	2	Explain the scientific background and rationale for the investigation being reported	6
Objectives	3	State specific objectives, including any prespecified hypotheses	7
**Methods**			
Study design	4	Present key elements of study design early in the paper	8
Setting	5	Describe the setting, locations, and relevant dates, including periods of recruitment, exposure, follow-up, and data collection	8
Participants	6	(*a*) Give the eligibility criteria, and the sources and methods of selection of participants	8
Variables	7	Clearly define all outcomes, exposures, predictors, potential confounders, and effect modifiers. Give diagnostic criteria, if applicable	9
Data sources/ measurement	8*	For each variable of interest, give sources of data and details of methods of assessment (measurement). Describe comparability of assessment methods if there is more than one group	*9*
Bias	9	Describe any efforts to address potential sources of bias	10
Study size	10	Explain how the study size was arrived at	8
Quantitative variables	11	Explain how quantitative variables were handled in the analyses. If applicable, describe which groupings were chosen and why	9
Statistical methods	12	(*a*) Describe all statistical methods, including those used to control for confounding	10
		(*b*) Describe any methods used to examine subgroups and interactions	9
		(*c*) Explain how missing data were addressed	11
		(*d*) If applicable, describe analytical methods taking account of sampling strategy	11
		(*e*) Describe any sensitivity analyses	8
**Results**			
Participants	13*	(a) Report numbers of individuals at each stage of study—eg numbers potentially eligible, examined for eligibility, confirmed eligible, included in the study, completing follow-up, and analysed	12
		(b) Give reasons for non-participation at each stage	NA
		(c) Consider use of a flow diagram	NA
Descriptive data	14*	(a) Give characteristics of study participants (eg demographic, clinical, social) and information on exposures and potential confounders	12
		(b) Indicate number of participants with missing data for each variable of interest	11
Outcome data	15*	Report numbers of outcome events or summary measures	13
Main results	16	(*a*) Give unadjusted estimates and, if applicable, confounder-adjusted estimates and their precision (eg, 95% confidence interval). Make clear which confounders were adjusted for and why they were included	13

For peer review only - http://bmjopen.bmj.com/site/about/guidelines.xhtml

		(*b*) Report category boundaries when continuous variables were categorized	13
		(*c*) If relevant, consider translating estimates of relative risk into absolute risk for a meaningful time period	NA
Other analyses	17	Report other analyses done—eg analyses of subgroups and interactions, and sensitivity analyses	13/14
**Discussion**			
Key results	18	Summarise key results with reference to study objectives	16
Limitations	19	Discuss limitations of the study, taking into account sources of potential bias or imprecision. Discuss both direction and magnitude of any potential bias	19
Interpretation	20	Give a cautious overall interpretation of results considering objectives, limitations, multiplicity of analyses, results from similar studies, and other relevant evidence	20
Generalisability	21	Discuss the generalisability (external validity) of the study results	21
**Other information**			
Funding	22	Give the source of funding and the role of the funders for the present study and, if applicable, for the original study on which the present article is based	2

*Give information separately for exposed and unexposed groups.

Note: An Explanation and Elaboration article discusses each checklist item and gives methodological background and published examples of transparent reporting. The STROBE checklist is best used in conjunction with this article (freely available on the Web sites of PLoS Medicine at http://www.plosmedicine.org/, Annals of Internal Medicine at http://www.annals.org/, and Epidemiology at http://www.epidem.com/). Information on the STROBE Initiative is available at www.strobe-statement.org.

For peer review only
